# Standardized LDH-to-lymphocyte ratio improves early mortality prediction in severe fever with thrombocytopenia syndrome: A 15-day competing-risk bedside model

**DOI:** 10.1371/journal.pntd.0014289

**Published:** 2026-04-27

**Authors:** Ruize Ma, Jingxia Wang, Zhouling Jiang, Shuwen Ding, Ruihua Zhang, Yanli Xu, Ranran Wang, Ling Lin, Zhihai Chen

**Affiliations:** 1 National Key Laboratory of Intelligent Tracking and Forecasting for Infectious Diseases, Beijing Ditan Hospital, Capital Medical University, Beijing, China; 2 Department of Infectious Diseases, Yantai Qishan Hospital (Yantai City Hospital for Infectious Disease), Yantai, China; JIHS National Institute of Infectious Diseases, JAPAN

## Abstract

**Background:**

Severe fever with thrombocytopenia syndrome (SFTS) is a tick-borne viral disease associated with a high mortality risk. Early triage is critical, but risk prediction can be biased because many patients are admitted several days after symptom onset and some leave hospital early.

**Methodology/Principal findings:**

We conducted a retrospective single-center cohort study of 459 consecutively hospitalized patients with laboratory-confirmed SFTS. The primary analysis included 392 patients with ascertained in-hospital vital status. Of these, 387 patients with complete admission predictor data formed the derivation cohort for a prespecified 15-day prediction horizon after symptom onset, selected to capture the clinically relevant early high-risk phase of SFTS. Symptom onset was treated as time zero, hospital admission as delayed entry (left truncation), and discharge alive within 15 days as a competing event. We compared the admission-based standardized lactate dehydrogenase-to-lymphocyte ratio (sLLR) with other ratio biomarkers for prediction of 15-day in-hospital death. We then developed a prespecified five-predictor bedside model including age, neurological manifestations, prothrombin time, platelet count, and sLLR. Individualized 15-day death risk was estimated as CIF@15 from cause-specific Cox models. Model performance was assessed by discrimination, calibration, clinical utility, and prediction error, with bootstrap internal validation. Among 387 patients in the derivation cohort, 67 died within 15 days. Admission sLLR showed the best discrimination for 15-day mortality (area under the curve [AUC] 0.797, 95% confidence interval [CI] 0.738–0.855). Using an ROC-derived threshold (sLLR ≥ 2.79), the 15-day cumulative incidence of in-hospital death was 48.2% versus 10.3% in the lower-sLLR group (P < 0.001). The five-predictor model improved discrimination (AUC 0.867, 95% CI 0.824–0.910) compared with the corresponding model without sLLR (P = 0.009), showed good calibration, provided higher net benefit across clinically relevant thresholds, and achieved low prediction error (Brier score at day 15: 0.097). sLLR added prognostic information beyond standard admission variables. A simple five-factor model may provide a practical tool for real-world early risk stratification of hospitalized patients with SFTS, helping clinicians identify those at increased risk of death within 15 days after symptom onset, although external validation in independent multicenter cohorts is still needed.

## Author summary

Severe fever with thrombocytopenia syndrome is a tick-borne infection that can be fatal. Clinicians need simple tools to identify high-risk patients early, but this is challenging because some patients arrive at hospital several days after symptoms begin, and some leave hospital before their outcome can be fully observed. We studied 459 hospitalized patients with laboratory-confirmed disease and focused on predicting death within 15 days after symptom onset. We found that a routine blood-test marker derived from lactate dehydrogenase and lymphocyte count—reflecting tissue injury and immune status—was strongly associated with early death. Patients with high values had a much higher probability of dying within 15 days than those with low values. We then combined this marker with a small set of readily available clinical variables to create a simple prediction model. This model provided more accurate and clinically useful risk estimates than models that did not include the marker. Our findings support a practical approach for early triage using routine admission information, which may help clinicians prioritize monitoring and supportive care for patients at highest risk.

## Introduction

Severe fever with thrombocytopenia syndrome (SFTS) is an acute tick-borne viral hemorrhagic illness caused by Dabie bandavirus (severe fever with thrombocytopenia syndrome virus, SFTSV) [1–3]. SFTS is associated with a high early mortality rate, and its clinical course often deteriorates rapidly within two weeks following symptom onset [4–6]. Most deaths are attributed to rapidly progressive multiple organ failure, which frequently involves the liver, kidneys, heart, and nervous system [7,8]. There is currently no vaccine for this disease, and no clear and unified antiviral treatment plan has been determined. The prognosis of patients largely depends on whether the disease progression can be identified early and supportive treatment can be provided in a timely manner [9]. The disease is prevalent in rural or mountainous areas where tertiary medical resources are limited [10]. There may be delays or misjudgments in the assessment of patients’ conditions upon admission, which may hinder timely triage and the upgrading of detection and supportive treatment. Therefore, simple, low-cost and easily accessible early risk stratification tools are still needed in clinical practice. In addition, in real-world settings, most patients with SFTS are individuals engaged in long-term outdoor activities, such as farmers and forestry workers [11], and most are elderly with relatively limited awareness of the disease [10,12]. In the early stage of symptom onset, some patients fail to seek medical treatment in time and often only enter the hospital for treatment after the condition has progressed. At the same time, with the improvement of supportive treatment, some patients with relatively mild conditions or faster recovery may be discharged from the hospital in a short period. This coexistence of delayed hospitalization and early discharge makes traditional prediction indicators and early warning models that take hospitalization time as the starting point and ignore the discharge outcome unable to accurately reflect the real short-term mortality risk, thereby affecting their application value in early triage and risk assessment [13].

From the perspective of pathogenic mechanisms, an increasing number of studies have shown that the severity of SFTS is not solely caused by the direct damage of the virus, but rather the result of the combined effect of viral replication and immune dysregulation. This is manifested as excessive inflammatory responses, vascular endothelial damage, coagulation dysfunction, and significant abnormalities in lymphocyte subsets [14–19]. In actual clinical diagnosis and treatment, severe or fatal cases often present with more significant lymphopenia, suggesting impaired immune function; at the same time, the level of lactate dehydrogenase (LDH) increases, reflecting extensive cellular and tissue damage [14,20]. Therefore, simultaneously considering these two indicators that contain implicit injury information may be more helpful in indicating the severity of the disease in its early stage and the risk of short-term adverse outcomes.

In other clinical settings, including COVID-19, related hyperinflammatory conditions such as MIS-A, HIV/AIDS-related hospitalization, and diffuse large B-cell lymphoma, LDH/lymphocyte-based ratios have also been reported to be associated with adverse outcomes [21–24]. Considering that both are routine tests for patients with SFTS upon clinical admission, and they are low-cost and easily accessible, the combined index of the two may have greater potential for promotion and bedside application.

In this study, we initially explored the value of the LDH/lymphocyte ratio in the early risk assessment of SFTS and constructed a standardized LDH/lymphocyte ratio (sLLR) for bedside risk stratification. Considering that patients often delay hospital admission and some are discharged within 15 days, we took the onset of the disease as the time zero point and focused on the 15-day window period after the onset of symptoms. This window was chosen because it captures the period during which early clinical deterioration and fatal progression are most likely to occur, and when triage decisions are most clinically consequential. We evaluated the sLLR and established a simple predictive model on the basis of handling delayed enrollment and competing discharge, to support early triage and supportive treatment decisions in endemic areas and resource-limited settings.

## Materials and methods

### Ethics statement

This study was led by Beijing Ditan Hospital, Capital Medical University, with Yantai Qishan Hospital serving as the participating and data-contributing site. The study was approved by the Ethics Committee of Beijing Ditan Hospital, Capital Medical University (No. DTEC-KY2022-022-01) and the Ethics Committee of Yantai Qishan Hospital (Approval No. 202513), and was conducted in accordance with the Declaration of Helsinki. Written informed consent was waived by the ethics committees because this retrospective study used only routinely collected clinical data, posed minimal risk to participants, and analyzed de-identified data.

### Study design and participants

We conducted a retrospective cohort study including patients with SFTS who were consecutively hospitalized at Yantai Qishan Hospital (Yantai, China) from January 2018 to December 2024. Laboratory confirmation of SFTS was based on any of the following criteria: (1) detection of SFTSV RNA in serum by RT-PCR; (2) a ≥ 4-fold increase in SFTSV-specific antibody titers or seroconversion in paired sera collected at an interval of ≥2 weeks; or (3) isolation of SFTSV from clinical samples. No patients in this cohort were included solely on the basis of viral isolation with negative RNA detection. The time of symptom onset was used as the time zero point. Because patients were admitted at different times after symptom onset, hospital admission was considered the entry point into the observation cohort (i.e., delayed entry/left truncation), and follow-up was subsequently handled using an onset-based time scale. The primary outcome window was defined as 15 days after symptom onset. This 15-day horizon was prespecified because prior studies have shown that severe progression and fatal outcomes in SFTS are concentrated within the first two weeks after symptom onset, making this period the most clinically relevant window for early triage and short-term risk assessment at admission [3,4,25,26].

Exclusion criteria were as follows: (i) lack of clear laboratory diagnostic evidence; (ii) confirmed co-infection with other viruses (such as hantavirus, dengue virus, Epstein–Barr virus [EBV], cytomegalovirus, etc.); (iii) missing preset time information or key predictive variables, making it impossible to complete time-to-event/prediction analysis; (iv) early exit from the index hospitalization due to transfer or self-discharge within the 15-day window, resulting in a heterogeneous and potentially informative follow-up process that could not be fully characterized using the available records; or (v) repeated hospitalizations, in which case only the first admission was retained. In the study database, such early transfer/self-discharge with unascertainable 15-day vital status was coded as Outcome = 3.

**Fig 1:**
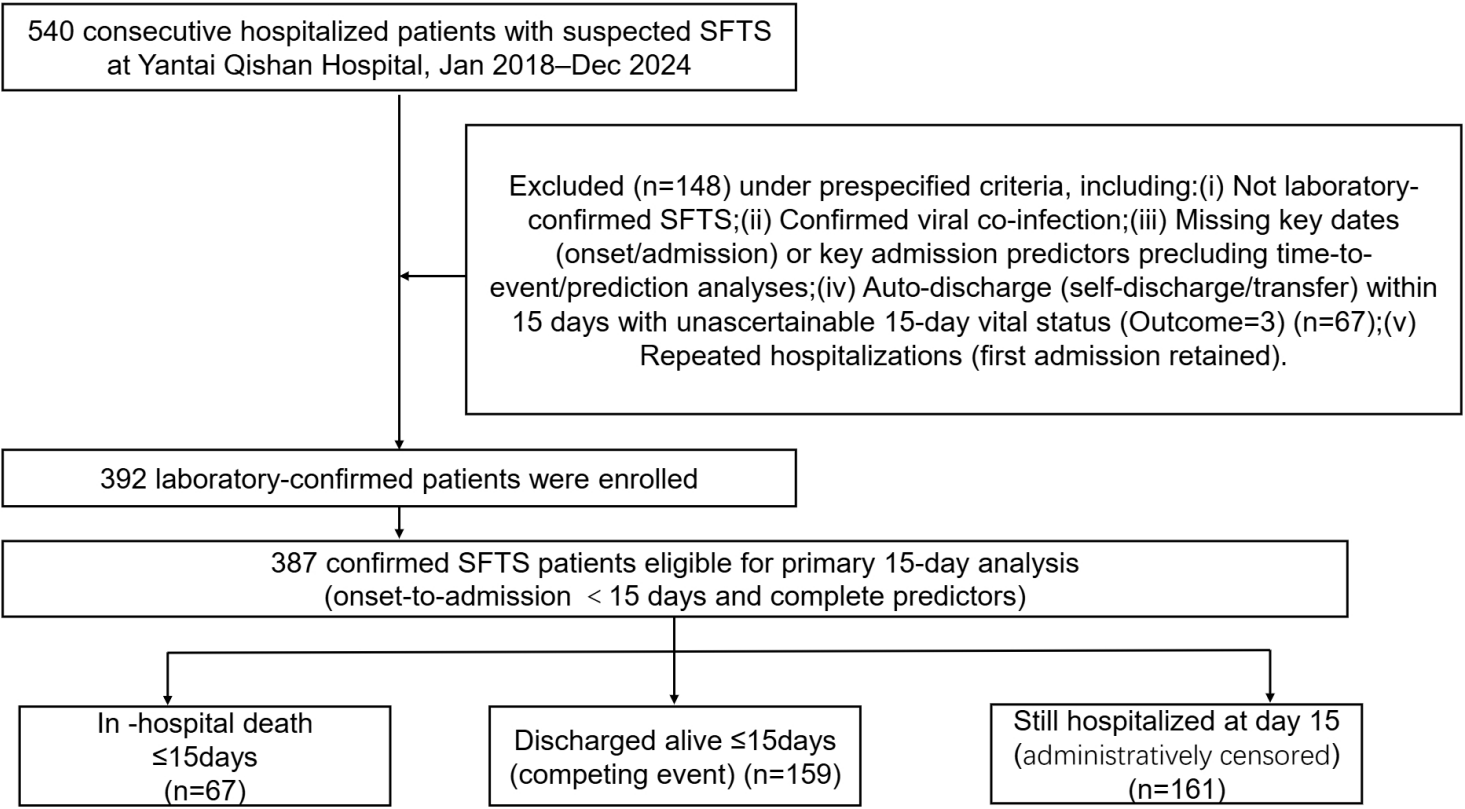
Flowchart of patient selection and outcomes within 15 days.

Among 540 consecutively hospitalized patients with suspected SFTS, 459 were laboratory-confirmed (Fig 1). Of these, 392 patients had ascertained in-hospital outcomes (Outcome = 0/1) and available admission viral load, whereas 67 patients were transferred or self-discharged with unascertainable post-discharge vital status (Outcome = 3) and had no viral-load record. The primary analysis excluded Outcome = 3 and was conducted in the 392 patients with ascertained outcomes. For the prespecified 15-day horizon, we further restricted to onset-to-admission <15 days and complete data for the bedside predictors, yielding an eligible analytic cohort of 387 patients. Outcome = 3 cases were incorporated in prespecified extreme-case sensitivity analyses (S3 Table; S1 Fig), and their descriptive characteristics are summarized in S10 Table.

Among 540 consecutively hospitalized patients with suspected SFTS (2018–2024), 81 were excluded under prespecified screening criteria, leaving 459 laboratory-confirmed cases. Of these, 392 had ascertained in-hospital vital status (Outcome = 0/1; viral load available), whereas 67 were transferred or self-discharged with unascertainable post-discharge vital status (Outcome = 3; viral load unavailable). The primary 15-day analysis included 387 patients with onset-to-admission <15 days and complete predictor data. Within 15 days after symptom onset, 67 patients died in hospital, 159 were discharged alive, and 161 remained hospitalized and were administratively censored at day 15. Outcome = 3 cases were incorporated in prespecified extreme-case sensitivity analyses (S3 Table; S1 Fig), and their descriptive characteristics are summarized in S10 Table.

### Definitions and outcomes

#### Onset definition.

Symptom onset was defined as the date when the first symptom compatible with SFTS was documented in the medical history. The onset-to-admission interval (Onset_to_Admission) was calculated as the number of days between symptom onset and hospital admission and was used to quantify delayed presentation.

#### Outcomes.

The primary outcome was in-hospital death within 15 days of symptom onset. Because patients entered observation only at admission, individuals were allowed to enter the risk set at hospital admission (delayed entry/left truncation), with entry time defined as Onset_to_Admission. Follow-up was administratively censored at day 15 after onset. Discharge alive within 15 days was treated as a competing event, and patients still hospitalized at day 15 were censored at that time. The estimand was the probability of in-hospital death by day 15 after onset among patients who survived to admission, accounting for delayed entry and competing discharge. Early transfer or self-discharge within 15 days with unascertainable post-discharge vital status was coded as Outcome = 3. These cases represent early non-routine exits (outcome missing) rather than confirmed discharge outcomes. Their descriptive characteristics and recorded exit times are summarized in S10 Table. As a supplementary analysis, we also described in-hospital outcomes occurring after the prespecified 15-day horizon among patients who remained hospitalized at day 15. In this landmark framework, day 15 after symptom onset was treated as the new time origin, and subsequent in-hospital death and discharge were summarized descriptively.

#### Variable definitions.

The primary exposure was the standardized lactate dehydrogenase-to-lymphocyte ratio (sLLR) at admission, calculated as lactate dehydrogenase (LDH, U/L) divided by the absolute lymphocyte count (×10⁹/L), and then rescaled by 1/1000 for reporting convenience. Because sLLR is a derived index rather than a routinely reported standalone clinical analyte, a universally established healthy reference interval is not currently available. To provide clinical context, the institutional admission reference ranges were 109–245 U/L for LDH and 1.1-3.2 × 10^9/L for absolute lymphocyte count. These component ranges provide only a reference background for interpreting sLLR and should not be regarded as a formally validated normal interval for the ratio itself. Importantly, the cutoff of 2.79 used in this study was a cohort-specific prognostic threshold derived from ROC analysis for 15-day mortality, rather than a normal upper reference limit. Admission SFTSV RNA viral load was quantified by RT-qPCR in the hospital laboratory when available, as previously described [27,28], expressed as copies/mL, and analyzed on a log10-transformed scale. All admission laboratory variables, including sLLR, were defined from the first blood sample obtained at hospital admission; therefore, the onset-to-sample interval was approximated by the onset-to-admission interval. Continuous predictors were entered with prespecified scaling to improve interpretability: age per 10 years, platelet count per 10 × 10⁹/L, prothrombin time per 1 s, and sLLR per 1 unit. For benchmarking, additional ratio indices derived from routine admission laboratory tests were calculated, including CAR, NLR, PLR, AAR, CLR, and UAR. Abbreviations, formulas, and units are provided in the Supporting information (S1 Table).

Neurological manifestations were defined as the documentation of at least one relevant symptom at admission, including headache, dizziness, impaired consciousness, apathy, bradyphrenia, somnolence, agitation, tremor, convulsions, or sluggish pupillary light reflex. Data were abstracted from admission notes using this prespecified definition. Hemorrhagic manifestations were defined as petechiae, purpura, or ecchymosis recorded at admission.

### Statistical analysis

The primary endpoint was in-hospital death within 15 days of onset. Time zero was symptom onset, and follow-up was administratively censored at day 15. Delayed entry (left truncation) was applied using the onset-to-admission interval. Death within 15 days was treated as the event of interest; discharge alive within 15 days was treated as a competing event; and patients still hospitalized at day 15 were censored at that time. Patients with transfer or self-discharge within 15 days (Outcome = 3) were excluded from the primary complete-case derivation set because early non-routine exits may introduce a potentially informative follow-up mechanism. Robustness was assessed by re-including these cases in prespecified extreme-case sensitivity analyses. Specifically, Outcome = 3 cases with recorded exit time ≤15 days were alternatively treated as (i) discharge alive (competing event) or (ii) death (worst-case scenario) at the recorded exit time, whereas Outcome = 3 cases with recorded exit time >15 days were administratively censored at day 15. Because complete pre-admission medication history was not systematically available, transfer from another hospital at admission was used as a pragmatic proxy for possible treatment exposure before admission. As a sensitivity analysis, the main analyses were repeated after excluding such patients. To address outcomes beyond the prespecified 15-day horizon, we performed a supplementary landmark analysis restricted to patients who remained hospitalized at day 15. In this analysis, time zero was reset to day 15 after symptom onset, and subsequent in-hospital death and discharge were described; cumulative incidence curves were compared by Gray’s test, and exploratory Cox models were fitted when the number of post-day-15 deaths permitted stable estimation. To evaluate whether the prognostic value of admission sLLR varied according to admission timing, we also performed prespecified subgroup analyses stratified by onset-to-admission interval (0–3 days, 4–7 days, and 8–14 days). Across these strata, we summarized admission sLLR distributions and 15-day event rates, and assessed discrimination using ROC/AUC analyses and association with 15-day mortality using cause-specific Cox models. An exploratory interaction analysis between sLLR and onset-to-admission strata was also performed.

For ROC/AUC analyses, the 15-day endpoint was operationalized as a binary outcome (death in hospital by day 15 vs. no such death), and this assessment was not intended as a time-dependent AUC under competing risks. The discriminatory ability of admission ratio indices was evaluated using ROC curves and AUCs. For comparability, AUCs and 95% confidence intervals were calculated on the same complete-case dataset, and paired DeLong tests were used to compare each index with sLLR. An optimal sLLR cut-off (2.79) was determined in the study cohort by maximizing the Youden index on the ROC curve for 15-day mortality, and patients were stratified accordingly. This pragmatic binary discrimination assessment was used solely for marker comparison and was conducted separately from the competing-risk modeling framework used to estimate CIF@15.

The 15-day cumulative incidence of in-hospital death after symptom onset was estimated using the cumulative incidence function (CIF), treating discharge alive within 15 days as a competing event; between-group differences were assessed using Gray’s test [29]. For descriptive visualization of sLLR-based risk stratification, CIF curves were plotted on the admission time scale with follow-up administratively censored at day 15 after symptom onset. A parsimonious admission prediction model was then constructed within the same onset-anchored time-to-event framework incorporating delayed entry (hospital admission as the entry time) and competing discharge. To clarify predictor specification and assess the incremental prognostic value of sLLR, we prespecified an admission model a priori—based on prognostic factors consistently reported in prior SFTS studies and established clinical relevance—rather than selecting predictors based on univariable significance; univariable analyses were descriptive and not used for predictor selection. Accordingly, we fitted cause-specific Cox proportional hazards models for (i) death and (ii) discharge, including age, neurological manifestations, prothrombin time, platelet count, and sLLR, and individualized 15-day death risk was estimated as CIF at day 15 (CIF@15). Model performance was evaluated at the prespecified 15-day horizon in terms of discrimination, calibration, clinical utility, and prediction error: discrimination was summarized using AUC, calibration was assessed using calibration plots at day 15, clinical utility was evaluated using decision-curve analysis (DCA) by estimating net benefit across threshold probabilities, and overall prediction error was quantified using the 15-day Brier score. Internal validation used bootstrap resampling to quantify uncertainty in performance measures. Statistical analyses were conducted in R (v4.4.2). Cause-specific Cox models were fitted using the survival package; cumulative incidence curves and Gray’s test were implemented using cmprsk; ROC analyses and paired DeLong tests were performed using pROC; and nomogram construction was performed using rms. Individualized CIF@15 prediction, calibration assessment, Brier score estimation, and decision-curve analysis were implemented using custom R functions based on the fitted cause-specific Cox models.

## Results

### Baseline characteristics of the study population

Among 540 consecutively hospitalized patients with suspected SFTS, 459 were laboratory-confirmed. Of these, 67 patients had early transfer or self-discharge with unascertainable post-discharge vital status (Outcome = 3) and were addressed in prespecified sensitivity analyses, leaving 392 patients with ascertained in-hospital outcomes for the primary analysis. For the prespecified 15-day horizon, we further restricted to patients admitted within 15 days after symptom onset with complete predictor data, yielding 387 eligible patients; 67 died in hospital within 15 days of symptom onset. The median time from symptom onset to admission was 5 days (IQR 4–7). Within the 15-day window, 159 patients were discharged alive, while 161 who remained hospitalized were administratively censored on day 15. For baseline comparisons, patients were categorized as having died within 15 days or not (Table 1).

**Table 1:** Selected baseline characteristics at admission by 15-day in-hospital mortality since symptom onset.

Variable	Overall (n = 387)	No in-hospital death by day 15 (n = 320)	Death within 15 days (n = 67)	P value
**Demographics**				
Female sex, n (%)	215 (55.6%)	181 (56.6%)	34 (50.7%)	0.384
Age, years†	66.00 (59.00–73.00)	65.00 (58.00–72.00)	71.00 (63.00–76.00)	<0.001
**Admission timing (delayed entry)**				
Onset-to-admission, days	5.00 (4.00–7.00)	5.00 (4.00–7.00)	5.00 (4.00–6.00)	0.660
**Comorbidities**				
Any comorbidity, n (%)	255 (65.9%)	215 (67.2%)	40 (59.7%)	0.240
**Key clinical feature**				
Neurological manifestations (any)†	188 (48.6%)	129 (40.3%)	59 (88.1%)	<0.001
**Key virological and model-related variables**				
Viral load, log10 copies/mL	3.27 (2.38–4.22)	2.99 (2.22–3.81)	4.45 (3.83–5.36)	<0.001
sLLR†	1.21 (0.66–2.38)	1.06 (0.60–1.97)	2.99 (1.71–4.56)	<0.001
LY	0.44 (0.31–0.69)	0.45 (0.32–0.70)	0.40 (0.26–0.52)	0.029
Platelet count (PLT)†	65.00 (49.00–85.00)	68.00 (52.00–88.00)	48.00 (36.00–67.00)	<0.001
LDH	531.00 (345.00–867.00)	470.50 (315.50–785.50)	900.00 (634.00–2128.93)	<0.001
CREA	68.00 (54.00–83.00)	66.00 (52.00–79.00)	79.70 (62.40–138.00)	<0.001
Prothrombin time (PT)†	13.00 (12.50–13.60)	13.05 (12.50–13.60)	13.00 (12.50–13.90)	0.778
APTT	48.10 (42.50–55.80)	47.20 (42.40–52.95)	60.30 (44.80–70.50)	<0.001
AST	129.80 (69.20–281.00)	117.50 (61.75–225.70)	282.30 (132.30–645.00)	<0.001
ALB	32.80 (29.50–35.80)	33.20 (30.10–36.15)	30.30 (27.40–33.30)	<0.001

**Notes:** Data are shown as n (%) for categorical variables and median (Q1–Q3) for continuous variables. Groups are defined by 15-day in-hospital mortality since symptom onset (time zero = symptom onset). The no in-hospital death group includes patients discharged alive within 15 days (n = 159) and those still hospitalized at day 15 (administratively censored; n = 161). All variables were assessed at admission unless otherwise specified. P values, if shown, were obtained using Pearson’s χ² test or Fisher’s exact test for categorical variables and the Wilcoxon rank-sum test for continuous variables; these comparisons are descriptive and were not used for predictor selection or causal inference. †Predictors included in the prespecified admission prediction model.

**Abbreviations:** sLLR, standardized lactate dehydrogenase-to-lymphocyte ratio; LY, lymphocyte count; LDH, lactate dehydrogenase; CREA, creatinine; PT, prothrombin time; APTT, activated partial thromboplastin time; AST, aspartate aminotransferase; ALB, albumin.

Compared with patients without in-hospital death by day 15, those who died within 15 days were older (median age, 71.0 vs 65.0 years; P < 0.001), whereas sex distribution and the prevalence of major pre-existing comorbidities were similar between groups. Neurological manifestations at admission were markedly more frequent in patients who died within 15 days (88.1% vs 40.3%; P < 0.001).

Patients who died within 15 days also had higher admission sLLR and viral load, together with lower lymphocyte and platelet counts. In addition, they showed a laboratory profile consistent with more severe early illness, including higher levels of LDH, creatinine, and AST, lower albumin levels, and longer activated partial thromboplastin time. By contrast, prothrombin time did not differ materially between groups.

### Admission sLLR and other routine ratios

**Fig 2:**
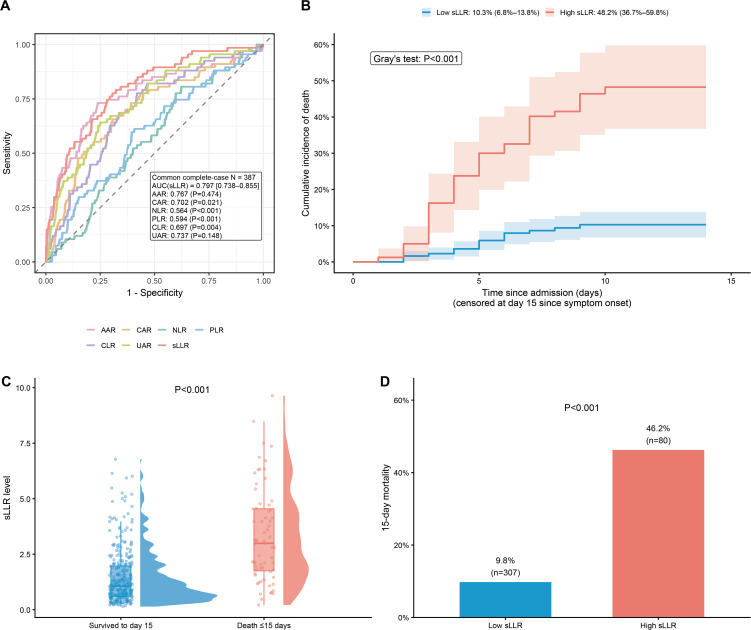
Discriminatory performance of sLLR and other admission biomarkers for 15-day mortality. It shows the discriminatory ability of sLLR and other routinely available admission-based ratio biomarkers for predicting in-hospital death within 15 days after symptom onset. (A) Receiver operating characteristic (ROC) curves comparing sLLR with CAR, NLR, PLR, AAR, CLR, and UAR for 15-day mortality. Areas under the curve (AUCs) were calculated on the same complete-case dataset, and pairwise comparisons with sLLR were performed using the DeLong test. (B) Cumulative incidence curves for in-hospital death within 15 days after symptom onset stratified by the optimal sLLR cut-off (2.79). Curves are shown on the admission time scale (time since admission) with delayed entry at hospital admission and administrative censoring at day 15; discharge alive within 15 days was treated as a competing event. Gray’s test was used to compare groups. Shaded areas indicate 95% confidence intervals. (C) Distribution of admission sLLR levels in patients who survived to day 15 versus those who died within 15 days; P values were calculated using the Wilcoxon rank-sum test. (D) Observed 15-day mortality by sLLR risk group (cut-off 2.79); P values were calculated using Fisher’s exact test or χ² test as appropriate.

At admission, sLLR was significantly higher in patients who died within 15 days (P < 0.001; Fig 2C). Among all routine ratio biomarkers evaluated, sLLR showed the highest discriminative ability for 15-day in-hospital mortality, with an AUC of 0.797 (95% CI 0.738–0.855; Fig 2A). Its performance was significantly superior to that of CAR, NLR, PLR, and CLR (all paired DeLong P < 0.05), while remaining comparable to AAR and UAR.

Using the ROC-cutoff of 2.79, crude 15-day mortality was 46.2% (37/80) in the high-sLLR group versus 9.8% (30/307) in the low-sLLR group (P < 0.001; Fig 2D). In competing-risk analyses, the 15-day cumulative incidence of in-hospital death was 48.2% in the high-sLLR group and 10.3% in the low-sLLR group (Gray’s test P < 0.001; Fig 2B).

### A prespecified five-predictor bedside model for early mortality risk stratification

**Fig 3:**
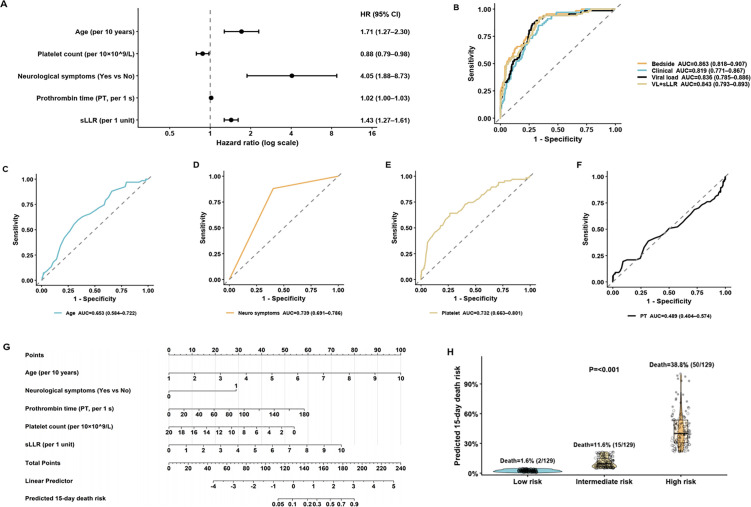
Development and clinical interpretation of the five-factor prediction model. It presents the construction, discrimination, and clinical interpretability of the prespecified five-factor prediction model for 15-day mortality. (A) Forest plot of the cause-specific Cox model for death with delayed entry at admission, including age (per 10 years), neurological manifestations (yes vs no), prothrombin time (per 1 s), platelet count (per 10 × 10^9/L), and sLLR (per 1 unit). (B–E) ROC curves for single-predictor models; discrimination was assessed by using each model’s predicted CIF@15 as the continuous risk score against observed death within 15 days (AUC with 95% CI). (F) ROC comparison of the clinical+sLLR model, the clinical-only model, and the sLLR-only model using predicted CIF@15; AUCs were compared with paired DeLong tests. (G) Nomogram for bedside estimation of 15-day in-hospital death risk. Each predictor is assigned points according to its value; the points are summed to generate a total score, which is then mapped to the predicted CIF@15. (H) Distribution of predicted CIF@15 across tertiles (low/intermediate/high) of the clinical+sLLR model.

We summarized the associations of routinely collected admission variables with 15-day in-hospital mortality using delayed-entry, cause-specific Cox models (Table 2, discharge model in S2 Table). Given the limited number of events (n = 67), to facilitate bedside application and avoid overfitting, we prespecified a small set of routine admission predictors a priori, informed by prior literature and clinical relevance. These predictors were selected to reflect key domains of SFTS severity—host factors, neurological involvement, coagulation function, thrombocytopenia, and tissue damage/immune suppression—while maintaining clinical interpretability and minimizing redundancy. This prespecified five-predictor model included age, neurological manifestations, prothrombin time (PT), platelet count, and sLLR; adjusted effects are shown in Fig 3A. In the complete-case modelling dataset (N = 387), all five predictors remained independently associated with early mortality: age per 10 years (HR 1.71, 95% CI 1.27–2.30; P < 0.001), neurological manifestations (HR 4.05, 95% CI 1.88–8.73; P < 0.001), PT per 1 s (HR 1.02, 95% CI 1.00–1.03; P = 0.039), and sLLR per 1 unit (HR 1.43, 95% CI 1.27–1.61; P < 0.001) increased risk, whereas platelet count was protective per 10 × 10^9/L (HR 0.88, 95% CI 0.79–0.98; P = 0.018). Admission viral load (log10) was strongly associated with 15-day mortality (univariable HR 2.17 per 1 log10, 95% CI 1.85–2.54; P < 0.001) and with a lower hazard of discharge (univariable HR 0.70, 95% CI 0.61–0.80; P < 0.001) (S2 Table). In an extended multivariable model that additionally included viral load alongside the five bedside predictors, viral load remained independently associated with mortality (HR 1.48, 95% CI 1.18–1.85; P < 0.001), while its association with discharge was attenuated (HR 0.85, 95% CI 0.71–1.01; P = 0.058) (S2 Table). To further characterize its practical impact, model performance with and without viral load is summarized in S4 Table, and bedside-model performance across viral-load tertiles is shown in S2 Fig. Details of cohort composition and outcome handling within the 15-day window are summarized in Fig 1, and robustness to early non-routine exits with unascertainable 15-day vital status was assessed in sensitivity analyses (S3 Table). Because complete pre-admission medication history was not systematically available, transfer from another hospital was used as a pragmatic proxy for possible treatment exposure before admission. Among patients in the 15-day derivation cohort, 45 had been transferred from other hospitals, whereas 342 were admitted directly. After excluding transferred patients, the main results remained directionally consistent: the AUC of sLLR changed from 0.797 to 0.786, and the AUC of the five-factor model changed from 0.867 to 0.870 (S7 Table).

**Table 2:** Cause-specific Cox model for death within 15 days since symptom onset Time origin was symptom onset (day 0). Admission was treated as delayed entry (left truncation at onset-to-admission), and follow-up was truncated at day 15 since onset. Discharge within 15 days was treated as a competing event; a cause-specific Cox model was fitted for death.

Variable	Univ HR (95% CI)	Univ P	Multiv (Bedside) HR (95% CI)	Multiv (Bedside) P	Multiv (Full) HR (95% CI)	Multiv (Full) P
Age (per 10 years)	1.649 (1.274–2.135)	<0.001	1.708 (1.272–2.295)	<0.001	1.743 (1.283–2.368)	<0.001
Neurological manifestations (Yes vs No)	8.587 (4.103–17.970)	<0.001	4.048 (1.877–8.732)	<0.001	3.264 (1.493–7.135)	0.003
Prothrombin time, PT (per 1 s)	1.032 (1.014–1.051)	<0.001	1.016 (1.001–1.031)	0.039	1.013 (0.998–1.029)	0.085
Platelet count, PLT (per 10 × 10 ^9^/L)	0.723 (0.646–0.811)	<0.001	0.878 (0.788–0.978)	0.018	0.925 (0.829–1.033)	0.166
sLLR (per 1 unit)	1.558 (1.408–1.725)	<0.001	1.431 (1.270–1.613)	<0.001	1.248 (1.082–1.440)	0.002
Viral load (log10, per 1)	2.169 (1.850–2.544)	<0.001			1.480 (1.184–1.851)	<0.001

**Notes:** HRs are cause-specific hazard ratios. Predictors were assessed at admission. Scaling: age per 10 years; PT per 1 s; platelet count per 10 × 10^9/L; sLLR per 1 unit; viral load per 1 log10. Analysis set: N = 387; death≤15 = 67; discharge≤15 = 159; administratively censored at day 15 (n = 161). Univariable models were descriptive and not used for predictor selection.

**Abbreviations:** HR, hazard ratio; CI, confidence interval; Univ, univariable cause-specific Cox model; Multiv, multivariable adjusted cause-specific Cox model; Bedside, prespecified five-predictor bedside model; Full, extended multivariable model additionally including viral load.

Discrimination of single predictors varied when evaluated for 15-day in-hospital mortality on the CIF@15 scale (Fig 3B–3E): age AUC 0.652 (95% CI 0.584–0.721), neurological manifestations AUC 0.761 (0.706–0.816), platelet count AUC 0.732 (0.665–0.799), and PT AUC 0.526 (0.451–0.601), indicating poor standalone discrimination for PT despite incremental information after adjustment. Combining predictors improved performance: the clinical+sLLR model achieved an AUC of 0.867 (95% CI 0.824–0.910), exceeding the clinical model without sLLR (AUC 0.823, 0.776–0.870; paired DeLong P = 0.009) and sLLR alone (AUC 0.800, 0.743–0.856; paired DeLong P = 0.007), whereas the clinical model and sLLR alone did not differ (P = 0.497) (Fig 3F). Performance estimates were robust to alternative assumptions regarding patients discharged/transferred within 15 days with unknown post-discharge vital status (S1 Fig; S3 Table). Specifically, the bedside model AUC@15 was 0.829 when Outcome = 3 cases were treated as deaths and 0.823 when treated as discharges (primary AUC 0.867), with Brier@15 ranging from 0.104 to 0.135 (S3 Table). To better understand the attenuation under the worst-case assumption, we additionally summarized the characteristics of patients with early transfer/self-discharge and unascertainable 15-day vital status (Outcome = 3) in S10 Table, including their recorded exit times and available admission clinical and laboratory variables. By definition, these patients had non-routine early exit from the index hospitalization, and viral-load data were unavailable for all cases. Their clinical and laboratory profiles suggested that this group was heterogeneous and could include patients with substantial disease severity at exit. Accordingly, reclassifying all such patients as deaths likely introduced a more severe and more uncertain case mix into the death group, which may explain the lower discrimination and higher prediction error observed under the worst-case scenario.

A nomogram derived from the five-predictor model facilitated bedside estimation of 15-day mortality risk (Fig 3G). To use the nomogram, each predictor value is located on its corresponding axis and projected upward to the points scale; the points are then summed across predictors, and the total score is mapped to the predicted cumulative incidence of in-hospital death by day 15 (CIF@15). For example, a patient aged 71 years with neurological manifestations, PT 13.9 s, platelet count 48 × 10^9/L, and sLLR 2.99 would accumulate a substantially higher total score on the nomogram, corresponding to a markedly increased predicted risk of death within 15 days. When stratified by tertiles of predicted risk (129 per group), observed 15-day mortality increased stepwise from 0.8% (1/129) to 12.4% (16/129) and 38.8% (50/129) in the low-, intermediate-, and high-risk groups, respectively (P < 0.001) (Fig 3H). Median predicted risks were 1.7% (IQR 1.0–2.6%), 7.8% (5.1–12.4%), and 34.0% (26.0–48.9%) across the three strata. Thus, in the present study, the highest tertile of predicted CIF@15 may be interpreted as a clinically high-risk group.

### Calibration, clinical utility, and prediction error for the 15-day horizons

**Fig 4:**
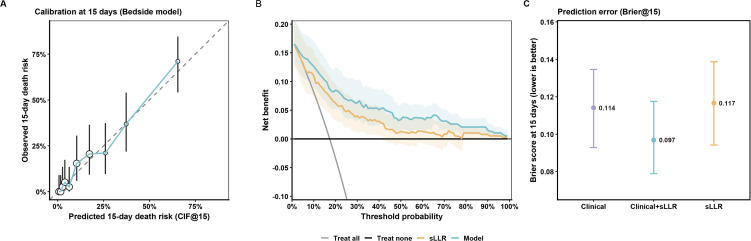
Calibration, clinical utility, and prediction error of the five-factor model. It evaluates the calibration, clinical usefulness, and overall prediction accuracy of the five-factor model for 15-day in-hospital mortality. (A) Calibration plot comparing predicted versus observed cumulative incidence of death at day 15 (CIF@15), with patients grouped by deciles of predicted risk. The dashed line represents perfect calibration. (B) Decision curve analysis (DCA) showing the net benefit of the prediction model across a range of threshold probabilities, compared with treat-all and treat-none strategies. (C) Prediction error summarized by the  Brier score at day 15, with points indicating estimates and vertical bars indicating 95% confidence intervals. Internal validation was performed using bootstrap resampling.

At the prespecified 15-day horizon after symptom onset, the clinical+sLLR model showed good agreement between predicted and observed risk on the CIF@15 scale (Fig 4A). Calibration points across deciles of predicted risk closely followed the 45° reference line, with modest underestimation in the highest risk decile (mean predicted 65.4% vs observed 71.1%). Decision-curve analysis supported clinical utility for early triage (Fig 4B): within the clinically relevant threshold-probability range of 0.09–0.60, the model yielded higher net benefit than both “treat-all” and “treat-none” strategies and consistently outperformed sLLR alone; using a conservative criterion based on the lower 95% CI, the model remained superior to both default strategies across 0.09–0.60, whereas sLLR alone did so over a narrower range (approximately 0.12–0.42). At a threshold probability of 0.60, net benefit was 0.036, corresponding to an estimated reduction of ~73.6 unnecessary interventions per 100 patients compared with a treat-all strategy. Overall prediction error at day 15 was lowest for the clinical+sLLR model (Brier@15 0.097, 95% CI 0.079–0.117), compared with the clinical model without sLLR (0.114, 0.094–0.133) and sLLR alone (0.117, 0.094–0.141) (Fig 4C). Sensitivity analyses addressing early non-routine exits and unascertainable 15-day vital status yielded broadly consistent discrimination and prediction error (S1 Fig; S3 Table).

As noted above, prespecified extreme-case sensitivity analyses incorporating Outcome = 3 cases yielded broadly similar conclusions, although discrimination was modestly attenuated and prediction error increased under the worst-case assumption (S3, S10 Tables; S1 Fig). Among patients with available viral load, bedside-model performance varied across viral-load tertiles, with AUC@15 decreasing from 0.944 (low VL) to 0.766 (high VL) as the event rate increased, and Brier@15 rising accordingly (S2 Fig).

### Additional analyses beyond day 15 and across onset-to-admission strata

To characterize outcomes beyond the prespecified 15-day horizon, we further evaluated the 161 patients who remained hospitalized at day 15. Among these landmark patients, only 6 later in-hospital deaths occurred, whereas 155 were subsequently discharged; the median additional follow-up beyond day 15 was 4 days (IQR 2–7). When stratified by the admission sLLR cut-off, later in-hospital death remained uncommon in both groups (4/130 [3.1%] in the low-sLLR group vs 2/31 [6.5%] in the high-sLLR group), with no clear difference in landmark cumulative incidence (Gray’s test P = 0.369). In exploratory landmark Cox analyses, admission sLLR was not clearly associated with later in-hospital death after day 15 (univariable HR 1.038, 95% CI 0.579–1.860; P = 0.900; multivariable HR 0.974, 95% CI 0.525–1.808; P = 0.934). These findings suggest that the prognostic utility of admission sLLR was concentrated mainly within the first 15 days after symptom onset, whereas later in-hospital deaths after day 15 were infrequent in this cohort.

We also examined whether the prognostic value of admission sLLR varied according to admission timing. Admission sLLR levels were broadly similar across onset-to-admission strata of 0–3 days, 4–7 days, and 8–14 days, with median values of 1.18 (IQR 0.70–2.10), 1.27 (0.67–2.40), and 1.04 (0.36–2.41), respectively (Kruskal–Wallis P = 0.331). The corresponding 15-day in-hospital mortality rates were 19.8% (16/81), 16.9% (44/261), and 15.6% (7/45). Across these strata, sLLR retained discriminative ability for 15-day mortality, with AUCs of 0.772 (95% CI 0.649–0.895), 0.791 (0.713–0.868), and 0.902 (0.803–1.000), respectively. In stratified cause-specific Cox analyses, higher admission sLLR remained associated with increased 15-day mortality in all three strata, with hazard ratios of 1.348 (95% CI 1.087–1.671; P = 0.006), 1.613 (1.428–1.821; P < 0.001), and 2.802 (1.553–5.053; P < 0.001), respectively. Because the 8–14 day stratum included relatively few patients and events, estimates in this subgroup should be interpreted cautiously. Detailed landmark and stratified results are provided in S8, S9 Tables and S4, S5 Figs.

## Discussion

SFTS often begins with nonspecific symptoms, mainly general manifestations such as fever, fatigue, and gastrointestinal discomfort, and may therefore not be recognized or taken seriously at an early stage. In endemic areas, many patients are older adults from rural populations, and limited awareness of the disease may further delay hospital presentation. At the same time, some patients with relatively mild illness may improve quickly and be discharged after a short period of treatment. These real-world features make early risk assessment difficult, and conventional admission-based biomarker studies might not consider the situation enough. To better reflect the actual clinical course of SFTS, we used symptom onset as time zero, allowed delayed entry at hospital admission, and treated discharge alive as a competing outcome. In this context, sLLR provided incremental prognostic information beyond the routine admission variables. A five-factor model including age, neurological symptoms, PT, platelet count, and sLLR showed stable performance in discrimination, calibration, decision curve, and prediction error analyses. The resulting CIF@15 estimates provide an interpretable measure of early mortality risk that may support triage, closer monitoring, and timely treatment planning for hospitalized patients with SFTS.

Several indicators derived from routine laboratory tests (e.g., CAR, CLR, NLR, PLR, AAR, and UAR) have been proposed for prognostic assessment and risk stratification in SFTS [30–34]. This issue is particularly relevant because SFTS often progresses rapidly during the first two weeks after symptom onset, when early clinical deterioration and in-hospital death are most likely to occur. However, reported cutoff values exhibit substantial inter-study heterogeneity, and the prognostic utility of these indices has demonstrated limited generalizability across diverse patient populations—highlighting the need for head-to-head comparison within a unified framework focused on the critical 15-day in-hospital window. While nomograms and multivariable models can provide more refined individualized risk estimates [35,36], some rely on numerous predictors or specialized assays such as quantitative viral load, limiting rapid implementation in primary care and resource-limited endemic settings where early triage is most urgent. In our cohort, admission viral load was markedly higher among patients who died within 15 days (Table 1) and remained independently associated with 15-day mortality in an extended multivariable model (Table 2), supporting its central role in early deterioration [28]. Nevertheless, because RT-qPCR-based viral-load quantification may not be immediately available at triage, and some transferred patients may not have comparable same-day viral-load data at the time of admission, we deliberately prioritized a parsimonious bedside model that does not require viral-load information while retaining strong prognostic performance. This model also remained stable across viral-load strata. These findings support the practical value of simple admission-based predictors for early risk stratification when quantitative viral-load testing is not readily available.

In the present study, sLLR is better understood as a marker of severe progression and short-term death risk in SFTS than as a disease-specific diagnostic marker. LDH is a simple and widely available marker of tissue injury, and higher LDH levels may reflect more severe organ damage and inflammation, including liver, kidney, or myocardial injury [20,37]. By contrast, lymphopenia reflects immune dysregulation and impaired host defense, which are common in severe or fatal SFTS [14,18,19,38,39]. Therefore, sLLR may capture both host injury and immune depletion at admission, rather than a single abnormality. This also helps explain its relation to viral load. In SFTS, high viral load is closely linked to cytokine storm, organ damage, coagulation disorder, and immune suppression. Viral load mainly reflects upstream pathogen burden, whereas sLLR may reflect the downstream host response to infection. This may explain why sLLR showed only moderate correlation with viral load in our cohort (S6 Table) and remained informative even without directly including viral load in the bedside model. Similar LDH/lymphocyte-based ratios have also been associated with lung involvement and poor outcomes in other acute viral illnesses [21,40], supporting the biological plausibility of this combined index. In severe SFTS, pulmonary infection and invasive pulmonary aspergillosis may further worsen prognosis [41,42]. Thus, sLLR may also help identify a high-risk clinical state related to both tissue injury and immune imbalance, although our data do not allow direct attribution of elevated sLLR to any specific pulmonary complication. Consistent with this interpretation, sensitivity analysis comparing the ratio formulation with modeling LDH and lymphocyte count as separate covariates favored the sLLR formulation in information criteria and predictive accuracy (S5 Table).

Within the first 15 days after symptom onset, sLLR showed the highest discriminative ability among the admission-based ratio markers evaluated in our cohort. One possible explanation is that early SFTS is often characterized by rapid clinical deterioration, and sLLR may reflect both tissue injury and immune disturbance. In comparison, some other ratio markers may capture more limited aspects of the disease process and may therefore be less informative in this early phase. Indicators such as CLR, NLR, and PLR may be more susceptible to the effects of secondary infections and treatments in the early stage and may fluctuate significantly within a short period of time. CAR reflects not only inflammation but also albumin-related factors such as nutritional status, liver synthetic function, and fluid dilution, which may reduce its stability across clinical settings [43]. AAR mainly reflects hepatocellular injury and may be influenced by underlying liver disease or drug-related liver injury, whereas UAR may reflect renal involvement and nutritional or volume status but is also affected by baseline renal function, dehydration, and fluid replacement. Therefore, in the assessment focused on the first 15 days after symptom onset, relying on a single inflammatory or organ-specific indicator may not adequately capture the key turning point in disease progression. This interpretation was also supported by sensitivity analysis, in which the ratio formulation outperformed modeling LDH and lymphocyte count as separate covariates in terms of information criteria and predictive accuracy (S5 Table). Combined with age, coagulation dysfunction, thrombocytopenia, and neurological manifestations, sLLR supports a simple admission-based model without requiring additional specialized tests, thereby facilitating early clinical decision-making and resource allocation.

The five-factor prediction model proposed in this study is clinically intuitive because each component reflects a key feature of early severe progression in SFTS. Age reflects a patient’s susceptibility and basic condition, and its association with adverse outcomes in SFTS is consistent. PT reflects early coagulation dysfunction and may indicate coagulation-factor consumption, hepatic impairment, and microcirculatory disturbance, all of which are linked to bleeding risk and organ dysfunction progression. Severe SFTS may progress to disseminated intravascular coagulation (DIC) [44], and previous studies have suggested that PT is associated with poor prognosis in SFTS. Thrombocytopenia is a core clinical feature of SFTS, and lower platelet counts are linked to higher bleeding risk and increased mortality during the acute phase [45–47]. Neurological symptoms at admission suggest early central nervous system involvement [8], which was strongly associated with death in our cohort and has also been reported in previous studies [48]. Together with sLLR, these variables form a simple admission-based model that captures vulnerability, coagulation dysfunction, bleeding tendency, immune–tissue injury burden, and early organ involvement, thereby supporting bedside risk stratification. Complete pre-admission medication history was not systematically available. However, review of the medical records suggested that most patients had received no major intervention before admission, and pre-admission treatment was usually limited to nonspecific symptomatic medications such as cold remedies, antipyretics, or antibiotics. The main concern was a minority of patients transferred from other hospitals after receiving treatments such as corticosteroids, hepatoprotective agents, or antiviral drugs. We therefore used transfer from another hospital as a pragmatic proxy for possible pre-admission treatment exposure and performed a sensitivity analysis excluding transferred patients. The main findings did not materially change, suggesting that the predictive performance of sLLR and the five-factor model was unlikely to be driven mainly by treatment-related distortion of admission laboratory values. Nevertheless, residual confounding related to treatment before admission cannot be fully excluded.

An important strength of this study is that the prediction framework was designed to reflect the actual clinical course of hospitalized SFTS. Because the interval from symptom onset to admission varies across patients, analyses that ignore delayed entry may distort the composition of the population at risk. In addition, within the first 15 days after symptom onset, discharge alive and in-hospital death are competing outcomes, and treating discharge as non-informative censoring may misrepresent the true risk of death. We therefore incorporated both delayed entry and competing discharge into model development and evaluation, so that CIF@15 can be directly interpreted as the cumulative risk of in-hospital death within 15 days after symptom onset. This framework is closely aligned with the early clinical course of SFTS and may support risk communication, monitoring, and resource allocation during early hospital management.

It should also be noted that, according to medical records, many patients discharged or transferred out of the hospital within 15 days, or whose outcomes could not be determined, were lost to follow-up due to their critical condition, leading their families to discontinue treatment or transfer them to another facility. Economic burden was an influencing factor in some cases. These factors may have introduced bias into the study sample. We therefore tested two extreme assumptions for these cases. In both scenarios, the overall pattern of risk stratification and the main conclusions remained similar. Model performance was weaker under the worst-case assumption, which is understandable because some of these early non-routine exits likely involved patients with substantial disease severity, including those whose families discontinued treatment, those transferred to higher-level hospitals for further care, and those affected by financial burden, while the subsequent outcomes were not fully documented in the medical records. Reclassifying all such patients as deaths would therefore increase the proportion of high-risk cases in the death group. Overall, these findings support the robustness of the main results, while also showing that early non-routine exits remain an important source of uncertainty in real-world prognostic modeling (S1 Fig; S3, S10 Tables).

This study has some limitations. First, the retrospective single-center design may limit generalizability and leaves the possibility of residual selection bias and unmeasured confounding. Second, the model was evaluated only with internal bootstrap validation. Although this approach supported discrimination, calibration, and overall prediction performance in the derivation cohort, external validation in independent cohorts is still required, and model recalibration may be needed across regions with different epidemic patterns, case mix, and levels of clinical care. Third, the present model was based mainly on information available at admission and did not account for dynamic changes during early hospitalization. Because SFTS can evolve rapidly during the early phase, incorporating serial clinical and laboratory measurements may further refine short-term prognostic assessment.

## Conclusion

The standardized LDH-to-lymphocyte ratio measured at admission may help identify hospitalized patients with SFTS who are at increased risk of death within 15 days after symptom onset. A simple model based on age, neurological symptoms, prothrombin time, platelet count, and sLLR showed good performance for early risk stratification in a clinically relevant framework that accounted for delayed admission and discharge alive. Although the findings were robust in sensitivity analyses, validation in independent multicenter cohorts is needed before routine clinical use.

## Supporting information

S1 Table Definitions and formulas of admission-based laboratory ratios. **Abbreviations:** sLLR, standardized lactate dehydrogenase-to-lymphocyte ratio; LDH, lactate dehydrogenase; CRP, C-reactive protein; CAR, C-reactive protein-to-albumin ratio; NLR, neutrophil-to-lymphocyte ratio; PLR, platelet-to-lymphocyte ratio; AAR, aspartate aminotransferase-to-alanine aminotransferase ratio; AST, aspartate aminotransferase; ALT, alanine aminotransferase; CLR, C-reactive protein-to-lymphocyte ratio; UAR, urea-to-albumin ratio. **Note:** sLLR is a derived biomarker based on LDH and absolute lymphocyte count and does not currently have a universally established reference interval for healthy individuals. In our hospital, the admission reference ranges were 109–245 U/L for LDH and 1.1–3.2 × 10^9/L for absolute lymphocyte count. These component ranges provide clinical context only and should not be interpreted as a formally validated reference interval for the ratio itself. The cutoff value of 2.79 in this study was derived for prognostic discrimination of 15-day in-hospital mortality and should not be interpreted as an upper limit of normal.
(DOCX)

S2 Table Cause-specific Cox model for discharge within 15 days since symptom onset. Time origin was symptom onset (day 0). Admission was treated as delayed entry (left truncation at onset-to-admission), and follow-up was truncated at day 15 since onset. Death within 15 days was treated as a competing event; a cause-specific Cox model was fitted for discharge. **Notes:** HRs are cause-specific hazard ratios. Predictors were assessed at admission. Scaling: age per 10 years; PT per 1 s; platelet count per 10 × 10^9/L; sLLR per 1 unit; viral load per 1 log10. Analysis set: N = 387; death≤15 = 67; discharge≤15 = 159; administratively censored at day 15 (n = 161). Univariable models were descriptive and not used for predictor selection. **Abbreviations:** HR, cause-specific hazard ratio; CI, confidence interval; PT, prothrombin time; PLT, platelet count; sLLR, standardized lactate dehydrogenase-to-lymphocyte ratio; Univ, univariable model; Multiv, multivariable model.
(DOCX)

S3 Table Sensitivity analyses for handling auto-discharge/transfer with unknown vital status (Outcome = 3). **Notes:** This table reports prespecified sensitivity analyses evaluating the robustness of model performance to alternative assumptions for patients with transfer or self-discharge and unascertainable post-discharge vital status (Outcome = 3). The primary model was developed and evaluated in the prespecified complete-case derivation cohort excluding Outcome = 3 (N = 387; Fig 1), defined by eligibility within the 15-day horizon (onset-to-admission <15 days) and complete predictors. Sensitivity analyses were conducted in an expanded cohort that added all Outcome = 3 cases (N = 67) to the 15-day–eligible Outcome = 0/1 cohort (N = 387), yielding a total N = 454; five laboratory-confirmed Outcome = 0/1 cases admitted on/after day 15 (onset-to-admission ≥15 days) were excluded because they contributed no at-risk time within the prespecified 15-day endpoint framework. Because the 15-day endpoint is defined from symptom onset, Outcome = 3 cases were handled based on their recorded exit time: those with exit time ≤15 days were reclassified under extreme assumptions as (SA-A) death or (SA-B) discharge alive at the recorded exit time, whereas Outcome = 3 cases with exit time >15 days were administratively censored at day 15. Discrimination and overall prediction error were summarized using AUC@15 and Brier@15, respectively. Overall, estimates remained broadly consistent across scenarios, supporting the robustness of the primary findings. Viral load was not available for Outcome = 3 cases and was therefore not incorporated in these sensitivity analyses.
(DOCX)

S4 Table Incremental value of viral load. Note: auto-discharge cases are Outcome = 3 and therefore not included in VL analyses. **Notes:** This table assesses the incremental prognostic value of admission SFTSV viral load beyond the prespecified five-predictor bedside model. Analyses were restricted to patients with available quantitative viral load measurements, and all models were compared on the same complete-case sample (N = 387; death≤15 = 67). Discrimination was evaluated using AUC for in-hospital death by day 15 after symptom onset, with paired DeLong tests used for model comparison. Overall prediction error was summarized using the 15-day Brier score (Brier@15). **Abbreviations:** AUC, area under the curve; Brier@15, Brier score at day 15; PT, prothrombin time; PLT, platelet count; sLLR, standardized lactate dehydrogenase-to-lymphocyte ratio.
(DOCX)

S5 Table Ratio validity: sLLR model vs a model including LDH and lymphocyte count as separate covariate. **Notes:** This table compares the ratio formulation (sLLR) with modelling LDH and lymphocyte count as separate covariates using the same complete-case dataset (N = 387). Model fit was assessed using AIC and BIC (lower values indicate better fit), and predictive performance was summarized by AUC for in-hospital death by day 15 and the 15-day Brier score (Brier@15). ΔAIC and ΔBIC denote differences between the component and ratio models. **Abbreviations:** sLLR, standardized lactate dehydrogenase-to-lymphocyte ratio; LDH, lactate dehydrogenase; AIC, Akaike information criterion; BIC, Bayesian information criterion; AUC, area under the curve; Brier@15, Brier score at day 15.
(DOCX)

S6 Table Spearman correlations between viral load (log10) and key bedside predictors. **Notes:** This table summarizes Spearman rank correlations between admission SFTSV viral load (log10) and key bedside predictors included in the prespecified model. Correlations were calculated using patients with available viral load measurements. Spearman’s ρ was used to assess monotonic associations without assuming linearity or normality. **Abbreviations:** sLLR, standardized lactate dehydrogenase-to-lymphocyte ratio; PT, prothrombin time; PLT, platelet count.
(DOCX)

S7 Table Sensitivity analysis after excluding patients transferred from other hospitals. **Notes:** Transfer from another hospital was used as a pragmatic proxy for possible pre-admission treatment exposure. Among the 387 patients in the 15-day derivation cohort, 45 (11.6%) were transferred from other hospitals. Excluding transferred patients did not materially change the discrimination of either sLLR or the five-factor model, supporting the robustness of the main findings. **Abbreviations:** AUC, area under the curve; CI, confidence interval; sLLR, standardized lactate dehydrogenase-to-lymphocyte ratio.
(DOCX)

S8 Table Landmark analysis of in-hospital outcomes after day 15 among patients still hospitalized at day 15. **Notes:** The primary analytic cohort comprised 387 patients with Outcome in {0,1}, onset-to-admission <15 days, and complete predictor data. Landmark analyses were restricted to the 161 patients who remained hospitalized at day 15 after symptom onset; in this subset, day 15 was treated as the new time origin. Panel A summarizes overall later outcomes. Panel B compares later in-hospital death after day 15 between admission sLLR groups defined using the prespecified cut-off of 2.79; the P value was obtained by Fisher’s exact test. Panel C shows exploratory landmark Cox models for later in-hospital death after day 15, with discharge treated as the competing clinical alternative. **Abbreviations:** sLLR, standardized lactate dehydrogenase-to-lymphocyte ratio; HR, hazard ratio; CI, confidence interval; IQR, interquartile range.
(DOCX)

S9 Table Stratified analyses of admission sLLR according to onset-to-admission interval. **Notes:** Onset-to-admission strata were prespecified as 0–3 days, 4–7 days, and 8–14 days. Panel A summarizes admission sLLR and observed outcomes in each stratum. Panel B reports ROC-based discrimination of admission sLLR for death within 15 days after symptom onset. Panel C reports univariable cause-specific Cox models for in-hospital death within 15 days, fitted on the onset-based time scale with delayed entry at admission and discharge alive within 15 days handled as the competing event. Estimates in the 8–14 day stratum should be interpreted cautiously because this subgroup included relatively few patients and events. **Abbreviations:** sLLR, standardized lactate dehydrogenase-to-lymphocyte ratio; AUC, area under the curve; HR, hazard ratio; CI, confidence interval; IQR, interquartile range.
(DOCX)

S10 Table Characteristics of patients with early transfer/self-discharge and unascertainable 15-day vital status (Outcome = 3). **Notes:** Outcome = 3 denotes early transfer or self-discharge with unascertainable 15-day vital status. The primary comparison cohort corresponds to the complete-case 15-day derivation cohort used for the main bedside model. Continuous variables are presented as median (Q1–Q3) and categorical variables as n (%). Subgroups of Outcome = 3 were defined according to recorded endpoint time since symptom onset (≤15 days vs > 15 days). **Abbreviations:** sLLR, standardized lactate dehydrogenase-to-lymphocyte ratio; LY, lymphocyte count; PLT, platelet count; LDH, lactate dehydrogenase; CREA, creatinine; PT, prothrombin time; APTT, activated partial thromboplastin time; AST, aspartate aminotransferase; ALB, albumin.
(DOCX)

S1 Fig Sensitivity analyses for handling early transfer/self-discharge with unascertainable 15-day vital status (Outcome = 3). (A) Discrimination of the prespecified bedside model summarized by AUC for in-hospital death by day 15 after symptom onset under three assumptions: primary analysis excluding Outcome = 3 within 15 days; SA-A treating Outcome = 3 within 15 days as death; and SA-B treating Outcome = 3 within 15 days as discharge. (B) Overall prediction error summarized by the 15-day Brier score (Brier@15; lower is better) under the same assumptions. Points indicate estimates and vertical bars indicate 95% confidence intervals. Analyses were performed in the expanded cohort including early non-routine exits.
(DOCX)

S2 Fig Bedside model performance across admission viral-load tertiles. (A) AUC for in-hospital death by day 15 after symptom onset for the prespecified bedside model within tertiles of admission SFTSV viral load (T1 low, T2 intermediate, T3 high). (B) Corresponding Brier@15 (lower is better). Points indicate estimates and vertical bars indicate 95% confidence intervals. Viral-load tertiles were defined among patients with available quantitative RT-qPCR results. Patients with early transfer/self-discharge and unascertainable 15-day vital status (Outcome = 3) were not included because viral-load measurements were unavailable for these cases.
(DOCX)

S3 Fig Incremental value of admission viral load beyond the prespecified bedside model. This figure graphically summarizes the comparisons reported in S4 Table. Admission SFTSV viral load was evaluated in relation to the prespecified five-predictor bedside model to assess whether adding viral load materially improved prediction of in-hospital death within 15 days after symptom onset. Discrimination was summarized using ROC/AUC-based comparisons on the same complete-case sample, and overall prediction error was evaluated using Brier@15. Viral-load analyses were restricted to patients with available quantitative RT-qPCR measurements; patients with early transfer/self-discharge and unascertainable 15-day vital status (Outcome = 3) were not included because viral-load data were unavailable for these cases.
(DOCX)

S4 Fig Landmark cumulative incidence of later in-hospital death after day 15 according to admission sLLR group. Landmark analyses were restricted to patients who remained hospitalized at day 15 after symptom onset, with day 15 treated as the new time origin. Curves show the cumulative incidence of later in-hospital death after the day-15 landmark stratified by the prespecified admission sLLR cut-off (2.79); discharge after day 15 was treated as the competing clinical alternative. Shaded areas indicate 95% confidence intervals. Gray’s test was used to compare groups.
(DOCX)

S5 Fig Admission sLLR across onset-to-admission strata and its association with 15-day mortality. (A) Distribution of admission sLLR values across prespecified onset-to-admission strata (0–3 days, 4–7 days, and 8–14 days). P values were calculated using the Kruskal–Wallis test. (B) Observed 15-day in-hospital mortality across the same onset-to-admission strata. Labels above bars indicate the mortality rate and stratum size. (C) ROC curves of admission sLLR for predicting in-hospital death within 15 days after symptom onset within each onset-to-admission stratum; AUCs with 95% confidence intervals are shown in the legend. These analyses were performed to assess whether the prognostic value of admission sLLR varied according to admission timing.
(DOCX)
