# Correction: Standardized LDH-to-lymphocyte ratio improves early mortality prediction in severe fever with thrombocytopenia syndrome: A 15-day competing-risk bedside model

**DOI:** 10.1371/journal.pntd.0014804

**Published:** 2026-10-16

**Authors:** Ruize Ma, Jingxia Wang, Zhouling Jiang, Shuwen Ding, Ruihua Zhang, Yanli Xu, Ranran Wang, Ling Lin, Zhihai Chen

There is an error in the Funding statement. The correct Funding statement is as follows: This work was supported by the National Key R&D Program of China (2022YFF1203201 to ZC). The funders had no role in study design, data collection and analysis, decision to publish, or preparation of the manuscript.
