# Supplementary material for: Standardized LDH-to-lymphocyte ratio improves early mortality prediction in severe fever with thrombocytopenia syndrome: A 15-day competing-risk bedside model
Source: PLoS Negl Trop Dis. 2026 Apr 27;20(4):e0014289. doi: 10.1371/journal.pntd.0014289 (PMC13138753; doi:10.1371/journal.pntd.0014289)
Supplement: S1 Table — Abbreviations: sLLR, standardized lactate dehydrogenase-to-lymphocyte ratio; LDH, lactate dehydrogenase; CRP, C-reactive protein; CAR, C-reactive protein-to-albumin ratio; NLR, neutrophil-to-lymphocyte ratio; PLR, platelet-to-lymphocyte ratio; AAR, aspartate aminotransferase-to-alanine aminotransferase ratio; AST, aspartate aminotransferase; ALT, alanine aminotransferase; CLR, C-reactive protein-to-lymphocyte ratio; UAR, urea-to-albumin ratio. Note: sLLR is a derived biomarker based on LDH and absolute lymphocyte count and does not currently have a universally established reference interval for healthy individuals. In our hospital, the admission reference ranges were 109–245 U/L for LDH and 1.1–3.2 × 10^9/L for absolute lymphocyte count. These component ranges provide clinical context only and should not be interpreted as a formally validated reference interval for the ratio itself. The cutoff value of 2.79 in this study was derived for prognostic discrimination of 15-day in-hospital mortality and should not be interpreted as an upper limit of normal. (DOCX) [file pntd.0014289.s001.docx]

**S1 Table. Definitions and formulas of admission-based laboratory ratios**

| Ratio | Full name | Formula |
| --- | --- | --- |
| sLLR | Standardized LDH-to-lymphocyte ratio | LDH (U/L) / absolute lymphocyte count (×10^9/L), rescaled by 1/1000 |
| CAR | CRP-to-albumin ratio | CRP / albumin |
| NLR | Neutrophil-to-lymphocyte ratio | Neutrophil / lymphocyte |
| PLR | Platelet-to-lymphocyte ratio | Platelet / lymphocyte |
| AAR | AST-to-ALT ratio | AST / ALT |
| CLR | CRP-to-lymphocyte ratio | CRP / lymphocyte |
| UAR | Urea-to-albumin ratio | Urea / albumin |

**Abbreviations:** sLLR, standardized lactate dehydrogenase-to-lymphocyte ratio; LDH, lactate dehydrogenase; CRP, C-reactive protein; CAR, C-reactive protein-to-albumin ratio; NLR, neutrophil-to-lymphocyte ratio; PLR, platelet-to-lymphocyte ratio; AAR, aspartate aminotransferase-to-alanine aminotransferase ratio; AST, aspartate aminotransferase; ALT, alanine aminotransferase; CLR, C-reactive protein-to-lymphocyte ratio; UAR, urea-to-albumin ratio.

**Note:** sLLR is a derived biomarker based on LDH and absolute lymphocyte count and does not currently have a universally established reference interval for healthy individuals. In our hospital, the admission reference ranges were 109–245 U/L for LDH and 1.1–3.2 ×10^9/L for absolute lymphocyte count. These component ranges provide clinical context only and should not be interpreted as a formally validated reference interval for the ratio itself. The cutoff value of 2.79 in this study was derived for prognostic discrimination of 15-day in-hospital mortality and should not be interpreted as an upper limit of normal.
