# Supplementary material for: Standardized LDH-to-lymphocyte ratio improves early mortality prediction in severe fever with thrombocytopenia syndrome: A 15-day competing-risk bedside model
Source: PLoS Negl Trop Dis. 2026 Apr 27;20(4):e0014289. doi: 10.1371/journal.pntd.0014289 (PMC13138753; doi:10.1371/journal.pntd.0014289)
Supplement: S2 Table — Time origin was symptom onset (day 0). Admission was treated as delayed entry (left truncation at onset-to-admission), and follow-up was truncated at day 15 since onset. Death within 15 days was treated as a competing event; a cause-specific Cox model was fitted for discharge. Notes: HRs are cause-specific hazard ratios. Predictors were assessed at admission. Scaling: age per 10 years; PT per 1 s; platelet count per 10 × 10^9/L; sLLR per 1 unit; viral load per 1 log10. Analysis set: N = 387; death≤15 = 67; discharge≤15 = 159; administratively censored at day 15 (n = 161). Univariable models were descriptive and not used for predictor selection. Abbreviations: HR, cause-specific hazard ratio; CI, confidence interval; PT, prothrombin time; PLT, platelet count; sLLR, standardized lactate dehydrogenase-to-lymphocyte ratio; Univ, univariable model; Multiv, multivariable model. (DOCX) [file pntd.0014289.s002.docx]

**S2 Table. Cause-specific Cox model for discharge within 15 days since symptom onset**

Time origin was symptom onset (day 0). Admission was treated as delayed entry (left truncation at onset-to-admission), and follow-up was truncated at day 15 since onset. Death within 15 days was treated as a competing event; a cause-specific Cox model was fitted for discharge.

| **Variable** | **Univ HR (95% CI)** | **Univ P** | **Multiv (Bedside) HR (95% CI)** | **Multiv (Bedside) P** | **Multiv (Full) HR (95% CI)** | **Multiv (Full) P** |
| --- | --- | --- | --- | --- | --- | --- |
| Age (per 10 years) | 0.892  (0.778–1.021) | 0.098 | 0.900 (0.779–1.040) | 0.153 | 0.909 (0.788–1.049) | 0.193 |
| Neurological manifestations (Yes vs No) | 0.485  (0.347–0.678) | <0.001 | 0.599 (0.421–0.854) | 0.005 | 0.639 (0.447–0.915) | 0.014 |
| Prothrombin time, PT (per 1 s) | 1.015  (0.989–1.042) | 0.256 | 1.032 (1.005–1.061) | 0.021 | 1.034 (1.006–1.063) | 0.018 |
| Platelet count, PLT (per 10×10^9/L) | 1.101  (1.043–1.161) | <0.001 | 1.032 (0.973–1.095) | 0.293 | 1.037 (0.977–1.100) | 0.234 |
| sLLR (per 1 unit) | 0.649  (0.546–0.772) | <0.001 | 0.681 (0.568–0.817) | <0.001 | 0.754 (0.616–0.923) | 0.006 |
| Viral load (log10, per 1) | 0.699  (0.610–0.801) | <0.001 |  |  | 0.846 (0.713–1.006) | 0.058 |

**Notes:** HRs are cause-specific hazard ratios. Predictors were assessed at admission. Scaling: age per 10 years; PT per 1 s; platelet count per 10×10^9/L; sLLR per 1 unit; viral load per 1 log10. Analysis set: N=387; death≤15=67; discharge≤15=159; administratively censored at day 15 (n=161). Univariable models were descriptive and not used for predictor selection.

**Abbreviations:** HR, cause-specific hazard ratio; CI, confidence interval; PT, prothrombin time; PLT, platelet count; sLLR, standardized lactate dehydrogenase-to-lymphocyte ratio; Univ, univariable model; Multiv, multivariable model.
