# Supplementary material for: Standardized LDH-to-lymphocyte ratio improves early mortality prediction in severe fever with thrombocytopenia syndrome: A 15-day competing-risk bedside model
Source: PLoS Negl Trop Dis. 2026 Apr 27;20(4):e0014289. doi: 10.1371/journal.pntd.0014289 (PMC13138753; doi:10.1371/journal.pntd.0014289)
Supplement: S3 Table — Notes: This table reports prespecified sensitivity analyses evaluating the robustness of model performance to alternative assumptions for patients with transfer or self-discharge and unascertainable post-discharge vital status (Outcome = 3). The primary model was developed and evaluated in the prespecified complete-case derivation cohort excluding Outcome = 3 (N = 387; Fig 1), defined by eligibility within the 15-day horizon (onset-to-admission <15 days) and complete predictors. Sensitivity analyses were conducted in an expanded cohort that added all Outcome = 3 cases (N = 67) to the 15-day–eligible Outcome = 0/1 cohort (N = 387), yielding a total N = 454; five laboratory-confirmed Outcome = 0/1 cases admitted on/after day 15 (onset-to-admission ≥15 days) were excluded because they contributed no at-risk time within the prespecified 15-day endpoint framework. Because the 15-day endpoint is defined from symptom onset, Outcome = 3 cases were handled based on their recorded exit time: those with exit time ≤15 days were reclassified under extreme assumptions as (SA-A) death or (SA-B) discharge alive at the recorded exit time, whereas Outcome = 3 cases with exit time >15 days were administratively censored at day 15. Discrimination and overall prediction error were summarized using AUC@15 and Brier@15, respectively. Overall, estimates remained broadly consistent across scenarios, supporting the robustness of the primary findings. Viral load was not available for Outcome = 3 cases and was therefore not incorporated in these sensitivity analyses. (DOCX) [file pntd.0014289.s003.docx]

**S3 Table. Sensitivity analyses for handling auto-discharge/transfer with unknown vital status (Outcome=3).**

| **Scenario** | **N** | **Death ≤15d** | **Discharge ≤15d** | **Event rate** | **AUC@15 (95% CI)** | **Brier@15 (95% CI)** |
| --- | --- | --- | --- | --- | --- | --- |
| Primary (exclude Outcome=3) | 387 | 67 | 159 | 0.173 | 0.867 (0.824–0.910) | 0.097 (0.079–0.117) |
| SA-A (Outcome=3; if T≤15 then treat as Death) | 454 | 114 | 159 | 0.251 | 0.829 (0.788–0.871) | 0.135 (0.116–0.154) |
| SA-B (Outcome=3; if T≤15 then treat as Discharge) | 454 | 67 | 206 | 0.148 | 0.823 (0.777–0.870) | 0.104 (0.086–0.123) |

**Notes: This table report**s prespecified sensitivity analyses evaluating the robustness of model performance to alternative assumptions for patients with transfer or self-discharge and unascertainable post-discharge vital status (Outcome=3). The primary model was developed and evaluated in the prespecified complete-case derivation cohort excluding Outcome=3 (N=387; Fig 1), defined by eligibility within the 15-day horizon (onset-to-admission <15 days) and complete predictors.

Sensitivity analyses were conducted in an expanded cohort that added all Outcome=3 cases (N=67) to the 15-day–eligible Outcome=0/1 cohort (N=387), yielding a total N=454; five laboratory-confirmed Outcome=0/1 cases admitted on/after day 15 (onset-to-admission ≥15 days) were excluded because they contributed no at-risk time within the prespecified 15-day endpoint framework.

Because the 15-day endpoint is defined from symptom onset, Outcome=3 cases were handled based on their recorded exit time: those with exit time ≤15 days were reclassified under extreme assumptions as (SA-A) death or (SA-B) discharge alive at the recorded exit time, whereas Outcome=3 cases with exit time >15 days were administratively censored at day 15. Discrimination and overall prediction error were summarized using AUC@15 and Brier@15, respectively. Overall, estimates remained broadly consistent across scenarios, supporting the robustness of the primary findings. Viral load was not available for Outcome=3 cases and was therefore not incorporated in these sensitivity analyses.
