# Supplementary material for: Standardized LDH-to-lymphocyte ratio improves early mortality prediction in severe fever with thrombocytopenia syndrome: A 15-day competing-risk bedside model
Source: PLoS Negl Trop Dis. 2026 Apr 27;20(4):e0014289. doi: 10.1371/journal.pntd.0014289 (PMC13138753; doi:10.1371/journal.pntd.0014289)
Supplement: S4 Table — Note: auto-discharge cases are Outcome = 3 and therefore not included in VL analyses. Notes: This table assesses the incremental prognostic value of admission SFTSV viral load beyond the prespecified five-predictor bedside model. Analyses were restricted to patients with available quantitative viral load measurements, and all models were compared on the same complete-case sample (N = 387; death≤15 = 67). Discrimination was evaluated using AUC for in-hospital death by day 15 after symptom onset, with paired DeLong tests used for model comparison. Overall prediction error was summarized using the 15-day Brier score (Brier@15). Abbreviations: AUC, area under the curve; Brier@15, Brier score at day 15; PT, prothrombin time; PLT, platelet count; sLLR, standardized lactate dehydrogenase-to-lymphocyte ratio. (DOCX) [file pntd.0014289.s004.docx]

**S4 Table. Incremental value of viral load. Note: auto-discharge cases are Outcome=3 and therefore not included in VL analyses.**

| Model | N | Death ≤15d | AUC (95% CI) | Brier@15 (95% CI) | ΔAUC (Full–Bedside) | DeLong P |
| --- | --- | --- | --- | --- | --- | --- |
| Bedside (Age + Neuro + PT + PLT + sLLR) | 387 | 67 | 0.867 (0.824–0.910) | 0.097 (0.079–0.117) | 0.020 | 0.02274 |
| Full (+ viral load) | 387 | 67 | 0.887 (0.848–0.926) | 0.090 (0.072–0.111) | 0.020 | 0.02274 |

**Notes:** This table assesses the incremental prognostic value of admission SFTSV viral load beyond the prespecified five-predictor bedside model. Analyses were restricted to patients with available quantitative viral load measurements, and all models were compared on the same complete-case sample (N=387; death≤15=67). Discrimination was evaluated using AUC for in-hospital death by day 15 after symptom onset, with paired DeLong tests used for model comparison. Overall prediction error was summarized using the 15-day Brier score (Brier@15).

**Abbreviations:** AUC, area under the curve; Brier@15, Brier score at day 15; PT, prothrombin time; PLT, platelet count; sLLR, standardized lactate dehydrogenase-to-lymphocyte ratio.
