# Supplementary material for: Standardized LDH-to-lymphocyte ratio improves early mortality prediction in severe fever with thrombocytopenia syndrome: A 15-day competing-risk bedside model
Source: PLoS Negl Trop Dis. 2026 Apr 27;20(4):e0014289. doi: 10.1371/journal.pntd.0014289 (PMC13138753; doi:10.1371/journal.pntd.0014289)
Supplement: S5 Table — Notes: This table compares the ratio formulation (sLLR) with modelling LDH and lymphocyte count as separate covariates using the same complete-case dataset (N = 387). Model fit was assessed using AIC and BIC (lower values indicate better fit), and predictive performance was summarized by AUC for in-hospital death by day 15 and the 15-day Brier score (Brier@15). ΔAIC and ΔBIC denote differences between the component and ratio models. Abbreviations: sLLR, standardized lactate dehydrogenase-to-lymphocyte ratio; LDH, lactate dehydrogenase; AIC, Akaike information criterion; BIC, Bayesian information criterion; AUC, area under the curve; Brier@15, Brier score at day 15. (DOCX) [file pntd.0014289.s005.docx]

**S5 Table. Ratio validity: sLLR model vs a model including LDH and lymphocyte count as separate covariate.**

| Model | N | AIC (death+discharge) | BIC (death+discharge) | AUC (95% CI) | Brier@15 (95% CI) | ΔAIC (B–A) | ΔBIC (B–A) |
| --- | --- | --- | --- | --- | --- | --- | --- |
| A: sLLR (ratio) | 387 | 2,366.3 | 2,405.9 | 0.867 (0.824–0.910) | 0.097 (0.079–0.117) | 14.9 | 22.8 |
| B: LDH + Lymphocyte (separate) | 387 | 2,381.2 | 2,428.7 | 0.852 (0.807–0.896) | 0.102 (0.085–0.122) | 14.9 | 22.8 |

**Notes:** This table compares the ratio formulation (sLLR) with modelling LDH and lymphocyte count as separate covariates using the same complete-case dataset (N=387). Model fit was assessed using AIC and BIC (lower values indicate better fit), and predictive performance was summarized by AUC for in-hospital death by day 15 and the 15-day Brier score (Brier@15). ΔAIC and ΔBIC denote differences between the component and ratio models.

**Abbreviations:** sLLR, standardized lactate dehydrogenase-to-lymphocyte ratio; LDH, lactate dehydrogenase; AIC, Akaike information criterion; BIC, Bayesian information criterion; AUC, area under the curve; Brier@15, Brier score at day 15.
