# Supplementary material for: Standardized LDH-to-lymphocyte ratio improves early mortality prediction in severe fever with thrombocytopenia syndrome: A 15-day competing-risk bedside model
Source: PLoS Negl Trop Dis. 2026 Apr 27;20(4):e0014289. doi: 10.1371/journal.pntd.0014289 (PMC13138753; doi:10.1371/journal.pntd.0014289)
Supplement: S6 Table — Notes: This table summarizes Spearman rank correlations between admission SFTSV viral load (log10) and key bedside predictors included in the prespecified model. Correlations were calculated using patients with available viral load measurements. Spearman’s ρ was used to assess monotonic associations without assuming linearity or normality. Abbreviations: sLLR, standardized lactate dehydrogenase-to-lymphocyte ratio; PT, prothrombin time; PLT, platelet count. (DOCX) [file pntd.0014289.s006.docx]

**S6 Table. Spearman correlations between viral load (log10) and key bedside predictors.**

| Predictor | Spearman ρ | P value |
| --- | --- | --- |
| sLLR | 0.687 | <0.0001 |
| PT | 0.067 | 0.1864 |
| PLT (per 10×10^9/L) | -0.366 | <0.0001 |
| Age (per 10 years) | 0.102 | 0.0441 |
| Neurological symptoms (Yes vs No) | 0.395 | <0.0001 |

**Notes:** This table summarizes Spearman rank correlations between admission SFTSV viral load (log10) and key bedside predictors included in the prespecified model. Correlations were calculated using patients with available viral load measurements. Spearman’s ρ was used to assess monotonic associations without assuming linearity or normality.

**Abbreviations:** sLLR, standardized lactate dehydrogenase-to-lymphocyte ratio; PT, prothrombin time; PLT, platelet count.
