# Supplementary material for: Standardized LDH-to-lymphocyte ratio improves early mortality prediction in severe fever with thrombocytopenia syndrome: A 15-day competing-risk bedside model
Source: PLoS Negl Trop Dis. 2026 Apr 27;20(4):e0014289. doi: 10.1371/journal.pntd.0014289 (PMC13138753; doi:10.1371/journal.pntd.0014289)
Supplement: S7 Table — Notes: Transfer from another hospital was used as a pragmatic proxy for possible pre-admission treatment exposure. Among the 387 patients in the 15-day derivation cohort, 45 (11.6%) were transferred from other hospitals. Excluding transferred patients did not materially change the discrimination of either sLLR or the five-factor model, supporting the robustness of the main findings. Abbreviations: AUC, area under the curve; CI, confidence interval; sLLR, standardized lactate dehydrogenase-to-lymphocyte ratio. (DOCX) [file pntd.0014289.s007.docx]

**S7 Table. Sensitivity analysis after excluding patients transferred from other hospitals.**

***Panel A. Cohort composition and 15-day outcomes by transfer status***

| Population | N | Transferred, n (%) | Direct admission, n (%) | 15-day deaths, n (%) |
| --- | --- | --- | --- | --- |
| Sheet4 main cohort | 392 | 46 (11.7) | 346 (88.3) | — |
| 15-day derivation cohort | 387 | 45 (11.6) | 342 (88.4) | 67 (17.3) |
| Direct-admission subgroup | 342 | 0 | 342 (100.0) | 60 (17.5) |
| Transferred subgroup | 45 | 45 (100.0) | 0 | 7 (15.6) |

***Panel B. Discrimination performance before and after excluding transferred patients***

| Analysis cohort | sLLR AUC (95% CI) | Five-factor model AUC (95% CI) |
| --- | --- | --- |
| Main derivation cohort (n=387; 67 deaths) | 0.797 (0.738–0.855) | 0.867 (0.824–0.911) |
| Exclude transferred patients (n=342; 60 deaths) | 0.786 (0.722–0.850) | 0.870 (0.825–0.916) |

**Notes:** Transfer from another hospital was used as a pragmatic proxy for possible pre-admission treatment exposure. Among the 387 patients in the 15-day derivation cohort, 45 (11.6%) were transferred from other hospitals. Excluding transferred patients did not materially change the discrimination of either sLLR or the five-factor model, supporting the robustness of the main findings.

**Abbreviations:** AUC, area under the curve; CI, confidence interval; sLLR, standardized lactate dehydrogenase-to-lymphocyte ratio.
