# Supplementary material for: Standardized LDH-to-lymphocyte ratio improves early mortality prediction in severe fever with thrombocytopenia syndrome: A 15-day competing-risk bedside model
Source: PLoS Negl Trop Dis. 2026 Apr 27;20(4):e0014289. doi: 10.1371/journal.pntd.0014289 (PMC13138753; doi:10.1371/journal.pntd.0014289)
Supplement: S8 Table — Notes: The primary analytic cohort comprised 387 patients with Outcome in {0,1}, onset-to-admission <15 days, and complete predictor data. Landmark analyses were restricted to the 161 patients who remained hospitalized at day 15 after symptom onset; in this subset, day 15 was treated as the new time origin. Panel A summarizes overall later outcomes. Panel B compares later in-hospital death after day 15 between admission sLLR groups defined using the prespecified cut-off of 2.79; the P value was obtained by Fisher’s exact test. Panel C shows exploratory landmark Cox models for later in-hospital death after day 15, with discharge treated as the competing clinical alternative. Abbreviations: sLLR, standardized lactate dehydrogenase-to-lymphocyte ratio; HR, hazard ratio; CI, confidence interval; IQR, interquartile range. (DOCX) [file pntd.0014289.s008.docx]

**S8 Table. Landmark analysis of in-hospital outcomes after day 15 among patients still hospitalized at day 15.**

***Panel A. Overview of outcomes within and beyond the prespecified 15-day horizon.***

| Cohort | N | Death ≤15 | Discharge ≤15 | Still hospitalized at day 15 | Death >15 | Discharge >15 | Additional follow-up beyond day 15, median (IQR), days |
| --- | --- | --- | --- | --- | --- | --- | --- |
| Primary analytic cohort | 387 | 67 | 159 | 161 | 6 | 155 | — |
| Landmark cohort (still hospitalized at day 15) | 161 | — | — | 161 | 6 | 155 | 4 (2-7) |

***Panel B. Later in-hospital death after day 15 according to admission sLLR group.***

| Admission sLLR group | N | Later in-hospital death, n/N (%) | Median admission sLLR | P value |
| --- | --- | --- | --- | --- |
| Low sLLR | 130 | 4/130 (3.1%) | 1.07 | 0.326 |
| High sLLR | 31 | 2/31 (6.5%) | 3.69 |  |

***Panel C. Landmark Cox analysis of admission sLLR for later in-hospital death after day 15.***

| Landmark model | HR (95% CI) for admission sLLR | P value |
| --- | --- | --- |
| Univariable landmark Cox | 1.038 (0.579-1.860) | 0.9 |
| Multivariable landmark Cox | 0.974 (0.525-1.808) | 0.934 |

**Notes:** The primary analytic cohort comprised 387 patients with Outcome in {0,1}, onset-to-admission <15 days, and complete predictor data. Landmark analyses were restricted to the 161 patients who remained hospitalized at day 15 after symptom onset; in this subset, day 15 was treated as the new time origin. Panel A summarizes overall later outcomes. Panel B compares later in-hospital death after day 15 between admission sLLR groups defined using the prespecified cut-off of 2.79; the P value was obtained by Fisher’s exact test. Panel C shows exploratory landmark Cox models for later in-hospital death after day 15, with discharge treated as the competing clinical alternative.

**Abbreviations:** sLLR, standardized lactate dehydrogenase-to-lymphocyte ratio; HR, hazard ratio; CI, confidence interval; IQR, interquartile range.
