# Supplementary material for: Standardized LDH-to-lymphocyte ratio improves early mortality prediction in severe fever with thrombocytopenia syndrome: A 15-day competing-risk bedside model
Source: PLoS Negl Trop Dis. 2026 Apr 27;20(4):e0014289. doi: 10.1371/journal.pntd.0014289 (PMC13138753; doi:10.1371/journal.pntd.0014289)
Supplement: S9 Table — Notes: Onset-to-admission strata were prespecified as 0–3 days, 4–7 days, and 8–14 days. Panel A summarizes admission sLLR and observed outcomes in each stratum. Panel B reports ROC-based discrimination of admission sLLR for death within 15 days after symptom onset. Panel C reports univariable cause-specific Cox models for in-hospital death within 15 days, fitted on the onset-based time scale with delayed entry at admission and discharge alive within 15 days handled as the competing event. Estimates in the 8–14 day stratum should be interpreted cautiously because this subgroup included relatively few patients and events. Abbreviations: sLLR, standardized lactate dehydrogenase-to-lymphocyte ratio; AUC, area under the curve; HR, hazard ratio; CI, confidence interval; IQR, interquartile range. (DOCX) [file pntd.0014289.s009.docx]

**S9 Table. Stratified analyses of admission sLLR according to onset-to-admission interval.**

***Panel A. Admission sLLR distribution and outcomes across onset-to-admission strata.***

| Onset-to-admission stratum | N | Admission sLLR, median (IQR) | 15-day in-hospital mortality, n/N (%) | Later in-hospital deaths (>15 d) | Later discharges (>15 d) |
| --- | --- | --- | --- | --- | --- |
| 0-3 days | 81 | 1.18 (0.70-2.10) | 16/81 (19.8%) | 0 | 21 |
| 4-7 days | 261 | 1.27 (0.67-2.40) | 44/261 (16.9%) | 3 | 104 |
| 8-14 days | 45 | 1.04 (0.36-2.41) | 7/45 (15.6%) | 3 | 30 |

***Panel B. Discrimination of admission sLLR for 15-day in-hospital mortality within each onset-to-admission stratum.***

| Onset-to-admission stratum | N | Deaths within 15 days | AUC (95% CI) |
| --- | --- | --- | --- |
| 0-3 days | 81 | 16 | 0.772 (0.649-0.895) |
| 4-7 days | 261 | 44 | 0.791 (0.713-0.868) |
| 8-14 days | 45 | 7 | 0.902 (0.803-1.000) |

***Panel C. Cause-specific Cox analyses of admission sLLR for 15-day in-hospital death within each onset-to-admission stratum.***

| Onset-to-admission stratum | N | Deaths within 15 days | HR (95% CI) for admission sLLR | P value |
| --- | --- | --- | --- | --- |
| 0-3 days | 81 | 16 | 1.348 (1.087-1.671) | 0.006 |
| 4-7 days | 261 | 44 | 1.613 (1.428-1.821) | <0.001 |
| 8-14 days | 45 | 7 | 2.802 (1.553-5.053) | <0.001 |

**Notes:** Onset-to-admission strata were prespecified as 0–3 days, 4–7 days, and 8–14 days. Panel A summarizes admission sLLR and observed outcomes in each stratum. Panel B reports ROC-based discrimination of admission sLLR for death within 15 days after symptom onset. Panel C reports univariable cause-specific Cox models for in-hospital death within 15 days, fitted on the onset-based time scale with delayed entry at admission and discharge alive within 15 days handled as the competing event. Estimates in the 8–14 day stratum should be interpreted cautiously because this subgroup included relatively few patients and events.

**Abbreviations:** sLLR, standardized lactate dehydrogenase-to-lymphocyte ratio; AUC, area under the curve; HR, hazard ratio; CI, confidence interval; IQR, interquartile range.
