# Supplementary material for: Standardized LDH-to-lymphocyte ratio improves early mortality prediction in severe fever with thrombocytopenia syndrome: A 15-day competing-risk bedside model
Source: PLoS Negl Trop Dis. 2026 Apr 27;20(4):e0014289. doi: 10.1371/journal.pntd.0014289 (PMC13138753; doi:10.1371/journal.pntd.0014289)
Supplement: S10 Table — Notes: Outcome = 3 denotes early transfer or self-discharge with unascertainable 15-day vital status. The primary comparison cohort corresponds to the complete-case 15-day derivation cohort used for the main bedside model. Continuous variables are presented as median (Q1–Q3) and categorical variables as n (%). Subgroups of Outcome = 3 were defined according to recorded endpoint time since symptom onset (≤15 days vs > 15 days). Abbreviations: sLLR, standardized lactate dehydrogenase-to-lymphocyte ratio; LY, lymphocyte count; PLT, platelet count; LDH, lactate dehydrogenase; CREA, creatinine; PT, prothrombin time; APTT, activated partial thromboplastin time; AST, aspartate aminotransferase; ALB, albumin. (DOCX) [file pntd.0014289.s010.docx]

**S10 Table. Characteristics of patients with early transfer/self-discharge and unascertainable 15-day vital status (Outcome=3).**

| Variable | Primary comparison cohort (Outcome=0/1, onset<15, complete predictors) | Outcome=3 overall | Outcome=3 recorded exit ≤15 days | Outcome=3 recorded exit >15 days |
| --- | --- | --- | --- | --- |
| N | 387 | 67 | 47 | 20 |
| Female sex, n (%) | 215 (55.6%) | 27 (40.3%) | 20 (42.6%) | 7 (35.0%) |
| Age, years | 66.00 (59.00–73.00) | 73.00 (67.00–78.00) | 74.00 (67.00–78.00) | 69.50 (66.75–74.75) |
| Onset-to-admission, days | 5.00 (4.00–7.00) | 5.00 (4.00–7.00) | 5.00 (3.00–7.00) | 7.00 (4.00–7.25) |
| Recorded endpoint time, days since symptom onset | 15.00 (12.00–18.00) | 12.00 (8.00–17.50) | 8.00 (7.00–12.00) | 20.50 (18.00–24.50) |
| Neurological manifestations, n (%) | 188 (48.6%) | 49 (73.1%) | 40 (85.1%) | 9 (45.0%) |
| sLLR | 1.21 (0.66–2.36) | 2.04 (0.96–3.27) | 2.05 (1.03–3.30) | 1.87 (0.76–3.17) |
| Lymphocyte count (LY) | 0.44 (0.31–0.69) | 0.51 (0.33–0.69) | 0.48 (0.33–0.67) | 0.54 (0.33–0.71) |
| Platelet count (PLT) | 65.00 (49.00–85.00) | 52.00 (40.00–74.50) | 52.00 (37.50–71.50) | 54.00 (43.75–79.75) |
| LDH | 531.00 (346.00–866.50) | 869.00 (443.50–1518.50) | 879.00 (478.50–1518.50) | 824.50 (362.00–1548.97) |
| Creatinine (CREA) | 68.00 (54.00–83.00) | 76.00 (59.05–99.00) | 80.00 (59.50–100.50) | 75.85 (58.50–95.75) |
| Prothrombin time (PT) | 13.00 (12.50–13.60) | 13.20 (12.60–13.80) | 13.20 (12.55–13.75) | 13.15 (12.60–13.80) |
| Activated partial thromboplastin time (APTT) | 48.10 (42.55–55.80) | 53.40 (47.75–65.45) | 56.60 (48.35–66.85) | 52.65 (47.55–57.03) |
| AST | 129.80 (69.25–279.35) | 198.40 (115.10–509.95) | 220.40 (123.85–538.75) | 140.50 (85.78–299.42) |
| Albumin (ALB) | 32.80 (29.50–35.80) | 30.70 (27.95–34.10) | 30.70 (27.80–34.20) | 30.50 (28.35–33.55) |
| Key bedside-predictor completeness, n/N (%) | Age=387/387 (100.0%); Neuro=387/387 (100.0%); PT=387/387 (100.0%); PLT=387/387 (100.0%); sLLR=387/387 (100.0%) | Age=67/67 (100.0%); Neuro=67/67 (100.0%); PT=67/67 (100.0%); PLT=67/67 (100.0%); sLLR=67/67 (100.0%) | Age=47/47 (100.0%); Neuro=47/47 (100.0%); PT=47/47 (100.0%); PLT=47/47 (100.0%); sLLR=47/47 (100.0%) | Age=20/20 (100.0%); Neuro=20/20 (100.0%); PT=20/20 (100.0%); PLT=20/20 (100.0%); sLLR=20/20 (100.0%) |

**Notes:** Outcome=3 denotes early transfer or self-discharge with unascertainable 15-day vital status. The primary comparison cohort corresponds to the complete-case 15-day derivation cohort used for the main bedside model. Continuous variables are presented as median (Q1–Q3) and categorical variables as n (%). Subgroups of Outcome=3 were defined according to recorded endpoint time since symptom onset (≤15 days vs >15 days).

**Abbreviations:** sLLR, standardized lactate dehydrogenase-to-lymphocyte ratio; LY, lymphocyte count; PLT, platelet count; LDH, lactate dehydrogenase; CREA, creatinine; PT, prothrombin time; APTT, activated partial thromboplastin time; AST, aspartate aminotransferase; ALB, albumin
