# Supplementary material for: Standardized LDH-to-lymphocyte ratio improves early mortality prediction in severe fever with thrombocytopenia syndrome: A 15-day competing-risk bedside model
Source: PLoS Negl Trop Dis. 2026 Apr 27;20(4):e0014289. doi: 10.1371/journal.pntd.0014289 (PMC13138753; doi:10.1371/journal.pntd.0014289)
Supplement: S1 Fig — (A) Discrimination of the prespecified bedside model summarized by AUC for in-hospital death by day 15 after symptom onset under three assumptions: primary analysis excluding Outcome = 3 within 15 days; SA-A treating Outcome = 3 within 15 days as death; and SA-B treating Outcome = 3 within 15 days as discharge. (B) Overall prediction error summarized by the 15-day Brier score (Brier@15; lower is better) under the same assumptions. Points indicate estimates and vertical bars indicate 95% confidence intervals. Analyses were performed in the expanded cohort including early non-routine exits. (DOCX) [file pntd.0014289.s011.docx]

**S1 Fig**


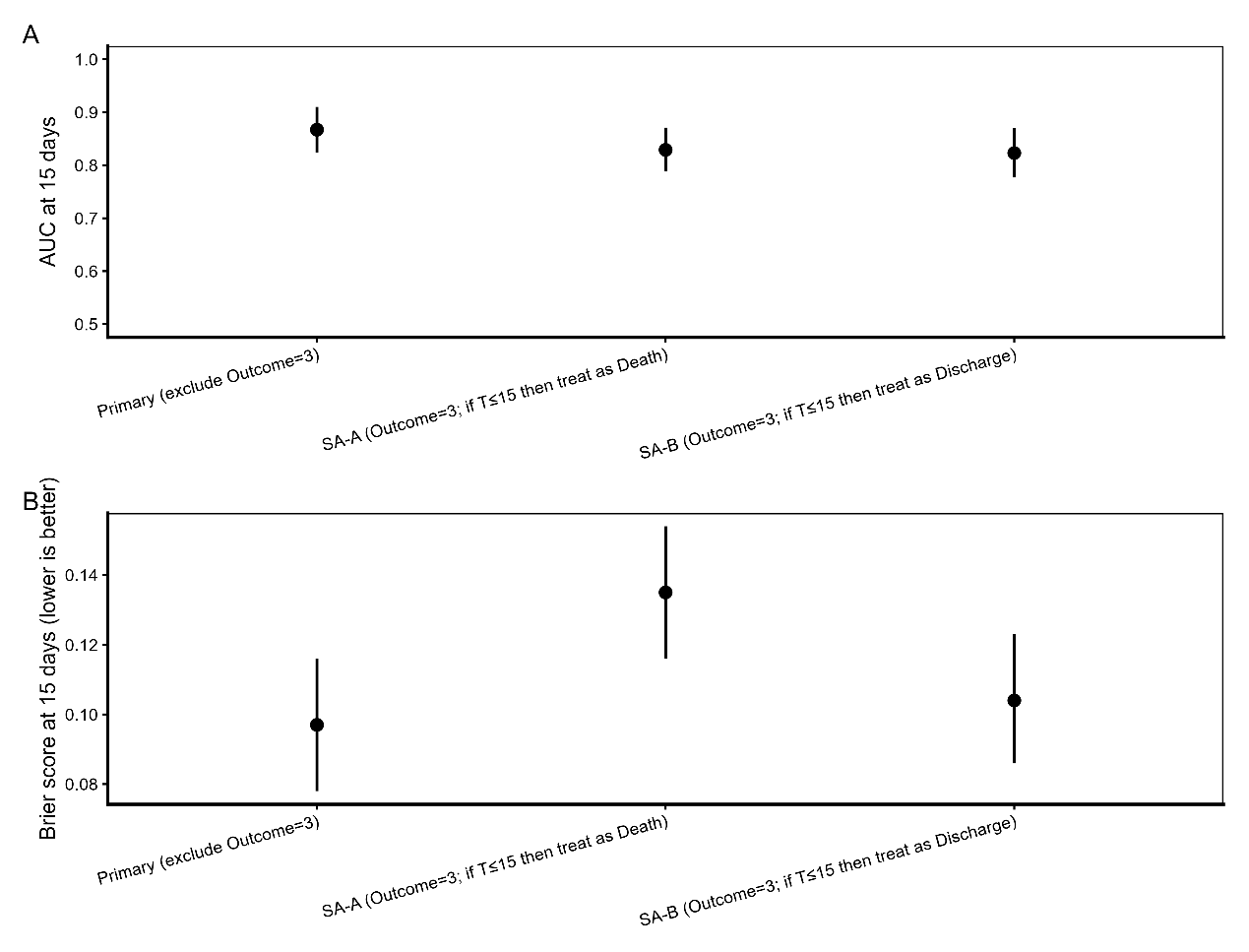


**S1 Fig. Sensitivity analyses for handling early transfer/self-discharge with unascertainable 15-day vital status (Outcome=3).**

(A) Discrimination of the prespecified bedside model summarized by AUC for in-hospital death by day 15 after symptom onset under three assumptions: primary analysis excluding Outcome=3 within 15 days; SA-A treating Outcome=3 within 15 days as death; and SA-B treating Outcome=3 within 15 days as discharge. (B) Overall prediction error summarized by the 15-day Brier score (Brier@15; lower is better) under the same assumptions. Points indicate estimates and vertical bars indicate 95% confidence intervals. Analyses were performed in the expanded cohort including early non-routine exits.
