# Supplementary material for: Standardized LDH-to-lymphocyte ratio improves early mortality prediction in severe fever with thrombocytopenia syndrome: A 15-day competing-risk bedside model
Source: PLoS Negl Trop Dis. 2026 Apr 27;20(4):e0014289. doi: 10.1371/journal.pntd.0014289 (PMC13138753; doi:10.1371/journal.pntd.0014289)
Supplement: S2 Fig — (A) AUC for in-hospital death by day 15 after symptom onset for the prespecified bedside model within tertiles of admission SFTSV viral load (T1 low, T2 intermediate, T3 high). (B) Corresponding Brier@15 (lower is better). Points indicate estimates and vertical bars indicate 95% confidence intervals. Viral-load tertiles were defined among patients with available quantitative RT-qPCR results. Patients with early transfer/self-discharge and unascertainable 15-day vital status (Outcome = 3) were not included because viral-load measurements were unavailable for these cases. (DOCX) [file pntd.0014289.s012.docx]

**S2 Fig**


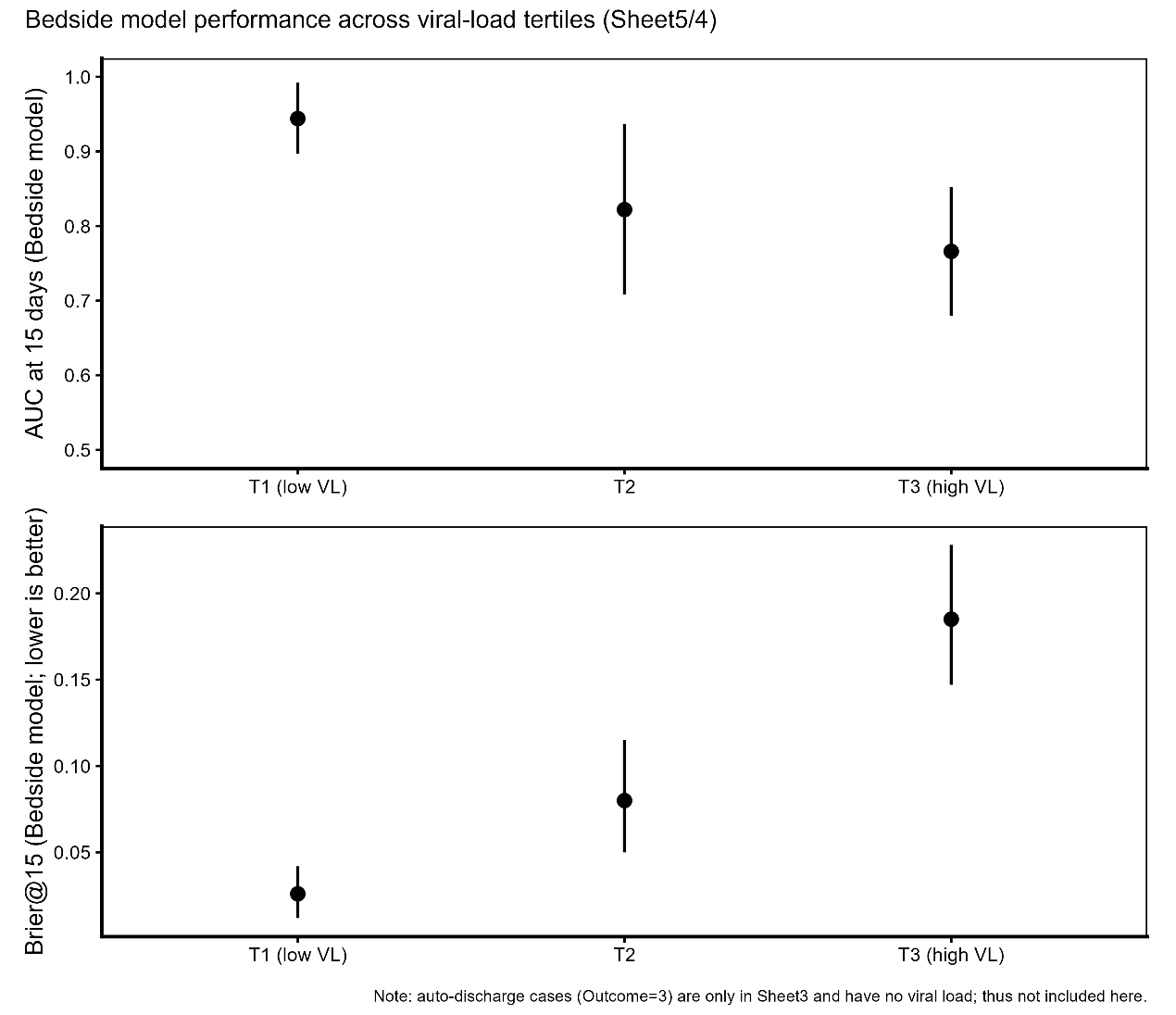


**S2 Fig. Bedside model performance across admission viral-load tertiles.**

(A) AUC for in-hospital death by day 15 after symptom onset for the prespecified bedside model within tertiles of admission SFTSV viral load (T1 low, T2 intermediate, T3 high). (B) Corresponding Brier@15 (lower is better). Points indicate estimates and vertical bars indicate 95% confidence intervals. Viral-load tertiles were defined among patients with available quantitative RT-qPCR results. Patients with early transfer/self-discharge and unascertainable 15-day vital status (Outcome=3) were not included because viral-load measurements were unavailable for these cases.
