# Supplementary material for: Standardized LDH-to-lymphocyte ratio improves early mortality prediction in severe fever with thrombocytopenia syndrome: A 15-day competing-risk bedside model
Source: PLoS Negl Trop Dis. 2026 Apr 27;20(4):e0014289. doi: 10.1371/journal.pntd.0014289 (PMC13138753; doi:10.1371/journal.pntd.0014289)
Supplement: S3 Fig — This figure graphically summarizes the comparisons reported in S4 Table. Admission SFTSV viral load was evaluated in relation to the prespecified five-predictor bedside model to assess whether adding viral load materially improved prediction of in-hospital death within 15 days after symptom onset. Discrimination was summarized using ROC/AUC-based comparisons on the same complete-case sample, and overall prediction error was evaluated using Brier@15. Viral-load analyses were restricted to patients with available quantitative RT-qPCR measurements; patients with early transfer/self-discharge and unascertainable 15-day vital status (Outcome = 3) were not included because viral-load data were unavailable for these cases. (DOCX) [file pntd.0014289.s013.docx]

**S3 Fig.**

**
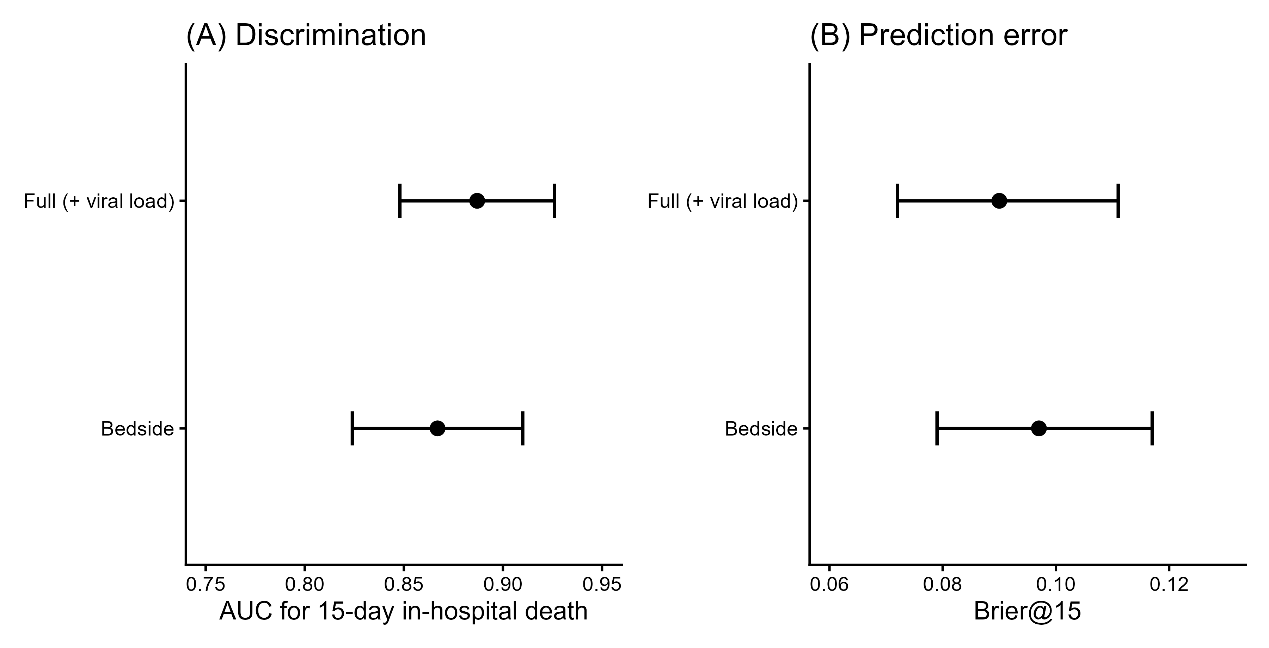
**

**S3 Fig. Incremental value of admission viral load beyond the prespecified bedside model.**

This figure graphically summarizes the comparisons reported in S4 Table. Admission SFTSV viral load was evaluated in relation to the prespecified five-predictor bedside model to assess whether adding viral load materially improved prediction of in-hospital death within 15 days after symptom onset. Discrimination was summarized using ROC/AUC-based comparisons on the same complete-case sample, and overall prediction error was evaluated using Brier@15. Viral-load analyses were restricted to patients with available quantitative RT-qPCR measurements; patients with early transfer/self-discharge and unascertainable 15-day vital status (Outcome=3) were not included because viral-load data were unavailable for these cases.
