# Supplementary material for: Standardized LDH-to-lymphocyte ratio improves early mortality prediction in severe fever with thrombocytopenia syndrome: A 15-day competing-risk bedside model
Source: PLoS Negl Trop Dis. 2026 Apr 27;20(4):e0014289. doi: 10.1371/journal.pntd.0014289 (PMC13138753; doi:10.1371/journal.pntd.0014289)
Supplement: S4 Fig — Landmark analyses were restricted to patients who remained hospitalized at day 15 after symptom onset, with day 15 treated as the new time origin. Curves show the cumulative incidence of later in-hospital death after the day-15 landmark stratified by the prespecified admission sLLR cut-off (2.79); discharge after day 15 was treated as the competing clinical alternative. Shaded areas indicate 95% confidence intervals. Gray’s test was used to compare groups. (DOCX) [file pntd.0014289.s014.docx]

S4 Fig.


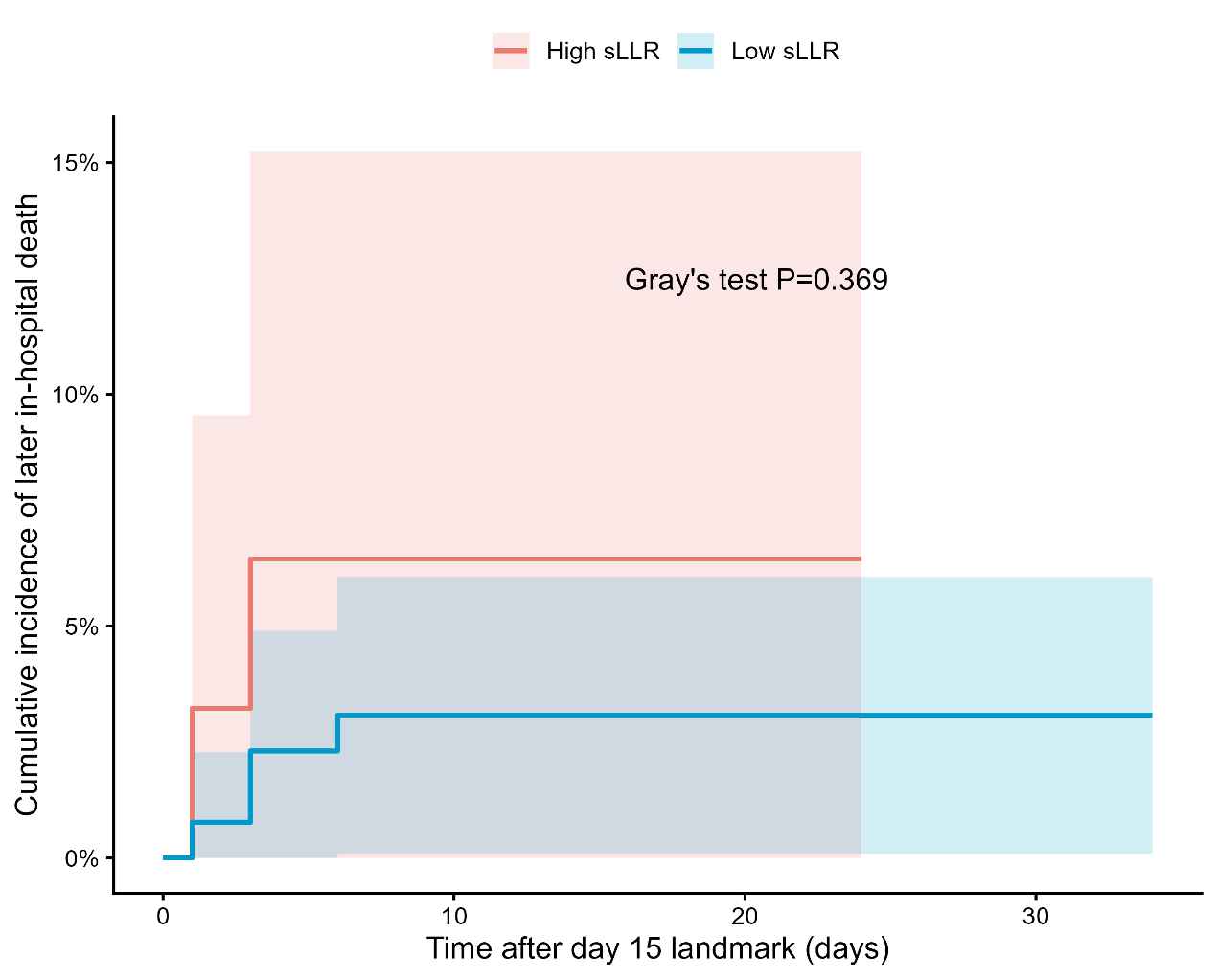


**S4 Fig. Landmark cumulative incidence of later in-hospital death after day 15 according to admission sLLR group.**

Landmark analyses were restricted to patients who remained hospitalized at day 15 after symptom onset, with day 15 treated as the new time origin. Curves show the cumulative incidence of later in-hospital death after the day-15 landmark stratified by the prespecified admission sLLR cut-off (2.79); discharge after day 15 was treated as the competing clinical alternative. Shaded areas indicate 95% confidence intervals. Gray’s test was used to compare groups.
