# Supplementary material for: Standardized LDH-to-lymphocyte ratio improves early mortality prediction in severe fever with thrombocytopenia syndrome: A 15-day competing-risk bedside model
Source: PLoS Negl Trop Dis. 2026 Apr 27;20(4):e0014289. doi: 10.1371/journal.pntd.0014289 (PMC13138753; doi:10.1371/journal.pntd.0014289)
Supplement: S5 Fig — (A) Distribution of admission sLLR values across prespecified onset-to-admission strata (0–3 days, 4–7 days, and 8–14 days). P values were calculated using the Kruskal–Wallis test. (B) Observed 15-day in-hospital mortality across the same onset-to-admission strata. Labels above bars indicate the mortality rate and stratum size. (C) ROC curves of admission sLLR for predicting in-hospital death within 15 days after symptom onset within each onset-to-admission stratum; AUCs with 95% confidence intervals are shown in the legend. These analyses were performed to assess whether the prognostic value of admission sLLR varied according to admission timing. (DOCX) [file pntd.0014289.s015.docx]

S5 Fig.


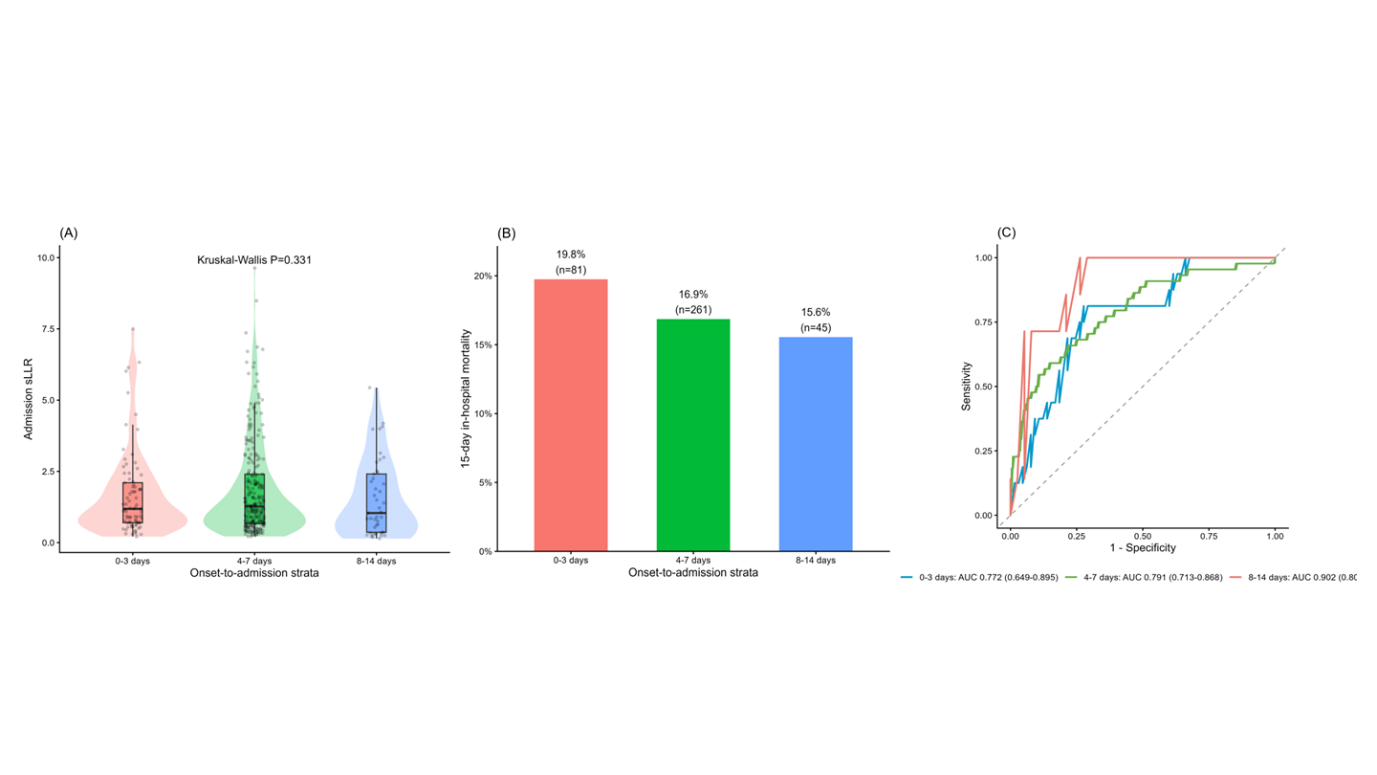


**S5 Fig. Admission sLLR across onset-to-admission strata and its association with 15-day mortality.**

(A) Distribution of admission sLLR values across prespecified onset-to-admission strata (0–3 days, 4–7 days, and 8–14 days). P values were calculated using the Kruskal–Wallis test.

(B) Observed 15-day in-hospital mortality across the same onset-to-admission strata. Labels above bars indicate the mortality rate and stratum size.

(C) ROC curves of admission sLLR for predicting in-hospital death within 15 days after symptom onset within each onset-to-admission stratum; AUCs with 95% confidence intervals are shown in the legend.

These analyses were performed to assess whether the prognostic value of admission sLLR varied according to admission timing.
